# Early tension regulation coupled to surface myomerger is necessary for the primary fusion of C2C12 myoblasts

**DOI:** 10.3389/fphys.2022.976715

**Published:** 2022-10-14

**Authors:** Madhura Chakraborty, Athul Sivan, Arikta Biswas, Bidisha Sinha

**Affiliations:** Department of Biological Sciences, Indian Institute of Science Education and Research Kolkata, Mohanpur, India

**Keywords:** myoblast fusion, membrane tension, membrane fluctuations, myomerger, cholesterol

## Abstract

Here, we study the time-dependent regulation of fluctuation–tension during myogenesis and the role of the fusogen, myomerger. We measure nanometric height fluctuations of the basal membrane of C2C12 cells after triggering differentiation. Fusion of cells increases fluctuation–tension but prefers a transient lowering of tension (at ∼2–24 h). Cells fail to fuse if early tension is continuously enhanced by methyl-β-cyclodextrin (MβCD). Perturbing tension regulation also reduces fusion. During this pre-fusion window, cells that finally differentiate usually display lower tension than other non-fusing cells, validating early tension states to be linked to fate decision. Early tension reduction is accompanied by low but gradually increasing level of the surface myomerger. Locally too, regions with higher myomerger intensity display lower tension. However, this negative correlation is lost in the early phase by MβCD-based cholesterol depletion or later as differentiation progresses. We find that with tension and surface-myomerger’s enrichment under these conditions, myomerger clusters become pronouncedly diffused. We, therefore, propose that low tension aided by clustered surface-myomerger at the early phase is crucial for fusion and can be disrupted by cholesterol-reducing molecules, implying the potential to affect muscle health.

## Introduction

Membrane lateral tension is used by cells to orchestrate diverse biological processes (Pontes et al., 2017). Even for membrane fusion, the mechanical state of the membrane is vital, as shown during the repair of lesions and cell–cell fusion (Demonbreun et al., 2015). The fusion of myoblasts to form myotubes is also an interesting problem with implications for human health. During the fusion of myoblasts to generate myotubes (termed myogenesis), studies across different model systems (Rochlin et al., 2010; Abmayr and Pavlath, 2012) implicate the regulation of cell mechanics. In insects, the tension of the actomyosin complex (cortical tension) is higher in myotubes and aids the fusion process. Membrane fluctuations, inversely proportional to the tension, have been proposed to function as a thermodynamic barrier preventing membrane–membrane interaction and fusion (Sharma, 2013). Once membranes hemi-fuse, completion of fusion again involves membrane stressing. Recent reports (Leikina et al., 2018) have demonstrated that artificially stressed or tensed membranes can functionally compensate for the lack of particular fusogens—such as myomerger—during myoblast fusion. Myomerger is one of the two proteins discovered in the last decade in vertebrate systems (Millay et al., 2014; Millay et al., 2016; Bi et al., 2017; Quinn et al., 2017; Leikina et al., 2018), playing a critical role in fusion. It is an 84aa single-pass protein, proposed to localize on the plasma membrane. It can generate positive spontaneous membrane curvature (Golani et al., 2021) and even indirectly stress the membrane, aiding the step from membrane hemi-fusion to complete fusion. Thus, membrane mechanics, especially membrane tension, may be expected to be regulated for successful cell–cell fusion during myogenesis. However, there are no systematic studies on the nature and need of membrane mechanical state during different stages of myogenesis to the best of our knowledge—which this work attempts to address.

What complicates the study is that cells display significant heterogeneity during myogenesis. Most cells retain their mononucleated form after many days of exposure to differentiation culture and co-exist with others that undergo fusion and terminal differentiation. These quiescent cells mimic satellite cells and successfully differentiate when cultured and exposed to differentiation conditions again (Yoshida et al., 1998). Therefore, we cannot neglect the possibility of different trajectories of membrane mechanics in these two kinds of cells—ones that differentiate and others that fail to differentiate. It is also possible that cells’ initial membrane mechanical states affect their ability to fuse. Deciphering and untangling the cell types require measurement of the membrane mechanical state in the same live cells as they go through differentiation. Therefore, the role of membrane mechanics, especially membrane’s fluctuation–tension, has been studied focusing on how membrane mechanics evolve, if it influences a cell’s fate/ability to differentiate, and how myomerger and tension inter-relate in differentiating cells.

In order to investigate this, we employed interference reflection microscopy (IRM) to measure fluctuations and derive information about effective membrane mechanical parameters, including membrane fluctuation–tension. The plasma membrane undergoes incessant shape or height (from substrate for the basal membrane) fluctuations due to the thermal background, and its fluctuation-amplitude and power-spectra are governed by its mechanical state. Tension derived from membrane fluctuations—termed fluctuation–tension—closely follows membrane frame tension and intrinsic membrane tension for a large range of tension values (Shiba et al., 2016). For brevity, membrane fluctuation–tension has been referred to as “tension” in the rest of the manuscript.

In this study, we assayed randomly selected cells in a population as well as followed single live cells for days after administrating the differentiation media (DM). To track the same cells for 5 days of differentiation, we used micropatterning to start with an aligned system and used marked dishes to find back the same regions on the different timepoints (days) of differentiation. Membrane mechanics were measured by IRM, while the surface level of myomerger was assayed in fixed samples *via* immunofluorescence (IF) and total internal reflection fluorescence microscopy (TIRF). When performed in tandem, IRM and widefield IF allowed correlative measurements of mechanics and fluorescence at the local scale and across cells. Together, this study provides new information about the alterations and coupling of tension and surface myomerger in cells undergoing cell–cell fusion in myogenesis.

## Results

### IRM imaging of differentiating C2C12 cells

**FIGURE 1:**
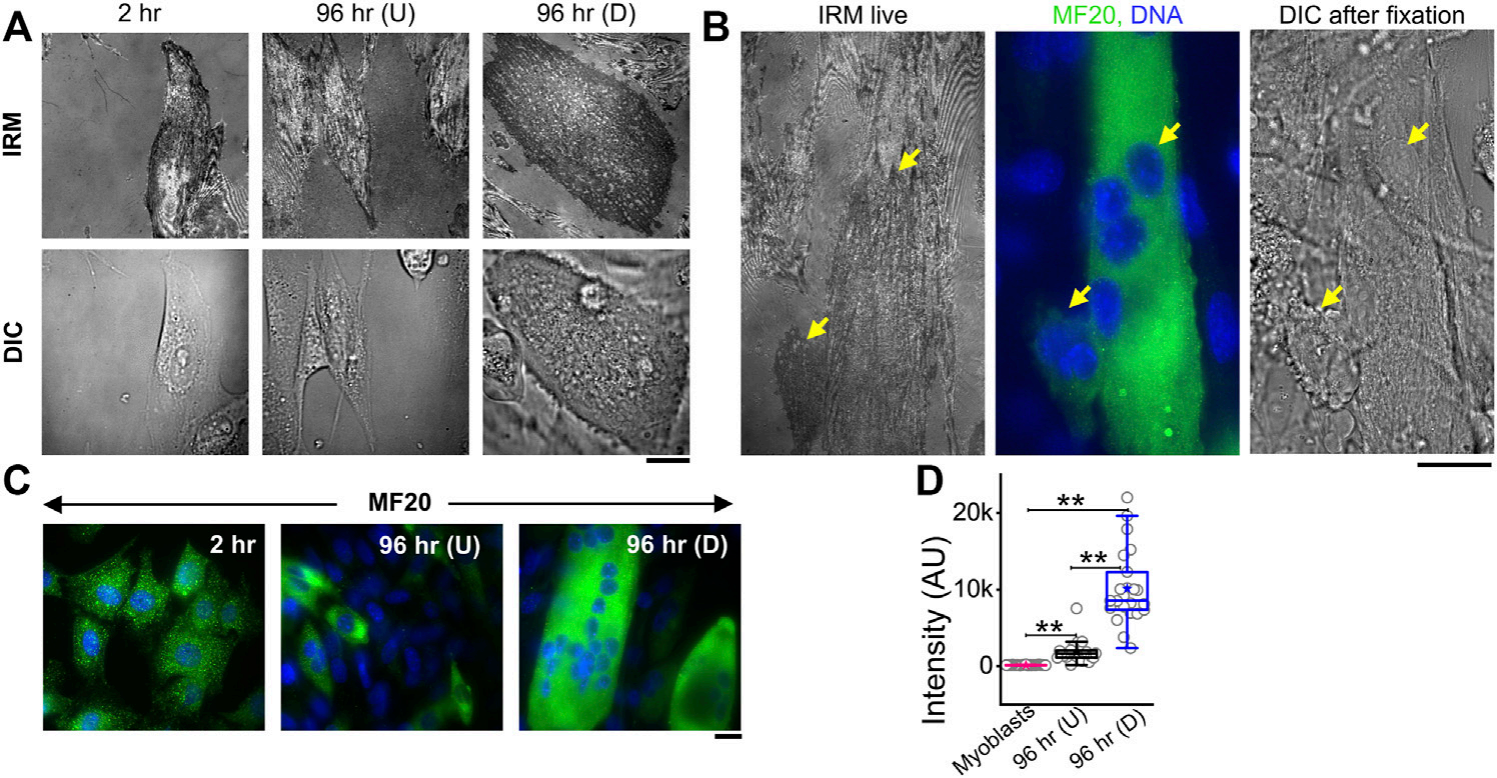
IRM and fluorescence images of C2C12 cells during differentiation. **(A)** Corresponding IRM and DIC images of C2C12 cells at different timepoints during the differentiation period. **(B)** Representative images of a multinucleated myotube marked by differentiation marker MF20 and DAPI for nucleic acid. Arrows point out myotube and single cell in live IRM and immunofluorescence and DIC after fixation. **(C)** Typical images of MF20 (immunostaining by anti MyHC antibody with goat anti-mouse Alexa fluor 488 secondary antibody) in three population of C2C12 cells. **(D)** Comparison of mean intensity of MF20 in cells; *n* = 3 independent experiments. “Myoblasts” indicate cells at 2 h post administration of differentiation media. 32 cells (2 h), 25 cells (96 hr U), and 24 cells (96 hr D) were used to get 1,565 (2 h), 626 (96 hr U), and 1,647 (96 hr D), respectively. In addition, 24 × 24 pixel regions were used in the plot. Scale bar = 20 µm. The Mann–Whitney U statistical significance test is performed; ** denotes *p* value < 0.001.

To measure the modulations of membrane mechanics during differentiation, C2C12 cells were imaged at various timepoints after administration of DM (Figure 1A, Supplementary Figure S1). As a control set, cells were imaged at 2 h post DM addition, ensuring maintaining the same refractive index of media for all timepoints—for better comparison of IRM. It is to be noted that it is known that no appreciable change in the gene expression profile starts by 2 h (Yoshida et al., 1998). New fusion events were observed to start from 48–96 h post DM, as expected. Hence, dishes were followed until the 96 h timepoint. At 96 h, cells having more than one nucleus—evident from DIC images of live cells—were noted as differentiated [96 hr (D)]. Since cells were expected to commit to differentiation by 96 h, unfused cells at 96 h were termed as undifferentiated [96 hr (U)]. Anti MyHC antibody staining (MF20), a known differentiation marker (Choi et al., 2020), was used to confirm differentiation and further validated that the multinucleated state and differentiation were correlated (Figure 1B). At 96 h, the mean MyHC intensity in differentiated cells is aproximately six times more than that of the undifferentiated cells (Figures 1C,D; Supplementary Table S1). The fusion index at 96 h was ∼30–50% whether the substrate was kept on a glass coverslip or PEG-coated glass micropatterned with adhesive line-shaped (20-μm wide and spaced 15 μm apart) patterns (Supplementary Figures S2A–E). Differentiated cells also showed a thicker cortical actin layer (Supplementary Figures S2F,G; Supplementary Table S1).

IRM images (Figures 1A,B) showed clear membrane topology alterations in differentiated cells. It should be noted that the intensity of IRM images indicates the relative distance between the basal membrane and the coverslip. The low intensity or dark regions arise due to this distance being minimized at focal adhesions or close attachment with the substrate. As the distance increases, the intensity increases until the first ∼100 nm—after which the intensity–height dependence reverses (Supplementary Figure S1). After differentiation, cells showed very uniform but darker intensities interspersed with lighter regions—unlike undifferentiated cells at 96 h or earlier timepoints. To the best of our knowledge, this work is the first report of IRM images of myogenesis. To understand if there were any robust patterns in these observations and if they indicated particular changes in membrane mechanics, next, the membrane height fluctuations were compared.

### Differentiation increases fluctuation–tension

**FIGURE 2:**
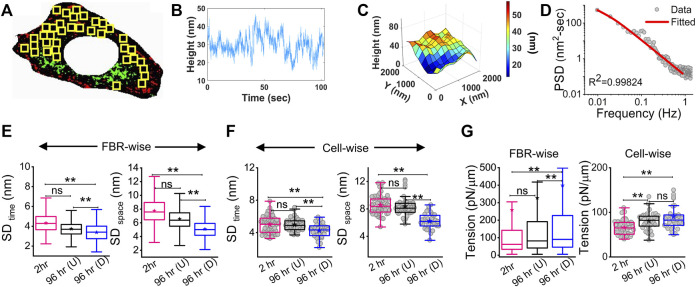
Change in membrane fluctuations and tension profile with time. **(A)** Typical composite image of a cell (red: IRM intensity minimas, green: IRM intensity maximas) with FBRs (12 × 12 pixels or 2.16 × 2.16 µm^2^) in yellow. **(B)** Representative profile of relative membrane height fluctuating over time at a single pixel. **(C)** Representative profile of relative membrane height undulations in space at a given timepoint. **(D)** Fitted PSD for a particular FBR with the R2 value of the fit indicated. **(E)** FBR-wise comparison of temporal and spatial fluctuation data among 2 hr, 96 hr (U) and 96 hr **(D)** cells; n = 3 independent experiments with 52 cells (2 hr), 40 cells (96 hr U), and 32 cells 96 hr **(D)** used to obtain 947, 712, and 948 FBRs (12 × 12 pixels), respectively. **(F)** Cell-wise temporal and spatial fluctuation profile of the same number of cells as mentioned in **(E)**. The effect of differentiation at 96 h has also been separately checked in three other independent experiments. **(G)** FBR and cell-wise comparison of tension; *n* = 3 and cell numbers are same as in E; number of 4 × 4 pixels (0.72 × 0.72 µm^2^) FBRs used are 14,269 (2 hr), 20,090 96 hr (U). and 35,041 96 hr (D). The linear mixed-effect model (LMM) was performed for FBR-wise comparisons. For others, the Mann–Whitney U statistical significance test was performed; ** denotes *p* value <0.001, * denotes *p* value <0.05, ns = not significant.

The membrane height fluctuations were calculated from the intensity changes by applying intensity–height conversion to pixels that were within ∼100 nm of the coverslip, falling in the first branch of the intensity–height calibration (Supplementary Figure S1D) (Biswas et al., 2017). These regions were termed as the first branch regions (FBRs) (yellow boxes, Figure 2A). FBRs were further filtered by avoiding the inclusion of pixels whose fluctuations were either very reduced (for example, at focal adhesions) or very high (usually in the cell interior) in the course of the complete time series (∼103 s). Multiple square regions were, thereby, identified where the intensity could be compared temporally or spatially to quantify height fluctuations.

There can be appreciable variability of membrane mechanics within single cells (Shi et al., 2018; Biswas et al., 2019). To capture local-level alterations in differentiation, FBRs from all cells of a particular condition were pooled and compared with other such pools (termed “FBR-wise”, Figure 2). The linear mixed-effect model (using the fixed effect of time and random effects grouped under replicate set-number of the experiments and cell number) was utilized to avoid the effect of the high sample size of FBRs influencing the hypothesis testing as also practiced for other high-sampling mechanical measurements (Herbig et al., 2018; Reichel et al., 2022). FBR-wise comparisons revealed how the distribution of local fluctuations/tension was affected by differentiation. To understand if there were global changes, cell-averaged quantities were pooled and compared (“cell-wise”, Figure 2).

Analysis of the fluctuations (Figures 2B–D) showed that for differentiated [96 hr (D)] cells, cell averages of temporal height fluctuations (SD_time_) and spatial undulations (SD_space_) were lower than those of the control (2 h) set or the cells which failed to differentiate [96h (U)] (Figure 2E; Supplementary Table S1). The reduction in the amplitude of spatial undulations found from comparing the height field across pixels in single FBRs of snapshots (SD_space_) (Figure 2F) corroborated the impression of a flatter membrane implicated by the uniform dark appearance of differentiated cells in IRM. Exponents calculated from the power spectral density (PSD) (Figure 2D) implied that cell membranes of differentiated cells were most “confined” (Supplementary Figure S3) (Brochard et al., 1975). Higher confinement and enhanced effective viscosity of the surrounding media were also reflected in the fit parameters obtained from fitting the PSD (Supplementary Figure S3) with the theoretical model.

The local tension distribution displayed a significant increase in the median on differentiation. However, cells that fail to differentiate in the same plates do not have such tension changes (Figure 2G; Supplementary Table S1). Although differentiated cells have significantly higher tension than cells at 2 h post DM addition and their cell-averaged tension was ∼10.5% more than 96 hr (U), the difference from the undifferentiated lot was statistically non-significant at the whole-cell level. We next visualize the distribution of tension in cells using tension maps (Biswas et al., 2019) and also check if local heterogeneities are enhanced for the observed increase in tension on differentiation.

### Spatial heterogeneity of fluctuation–tension increases with the mean

**FIGURE 3:**
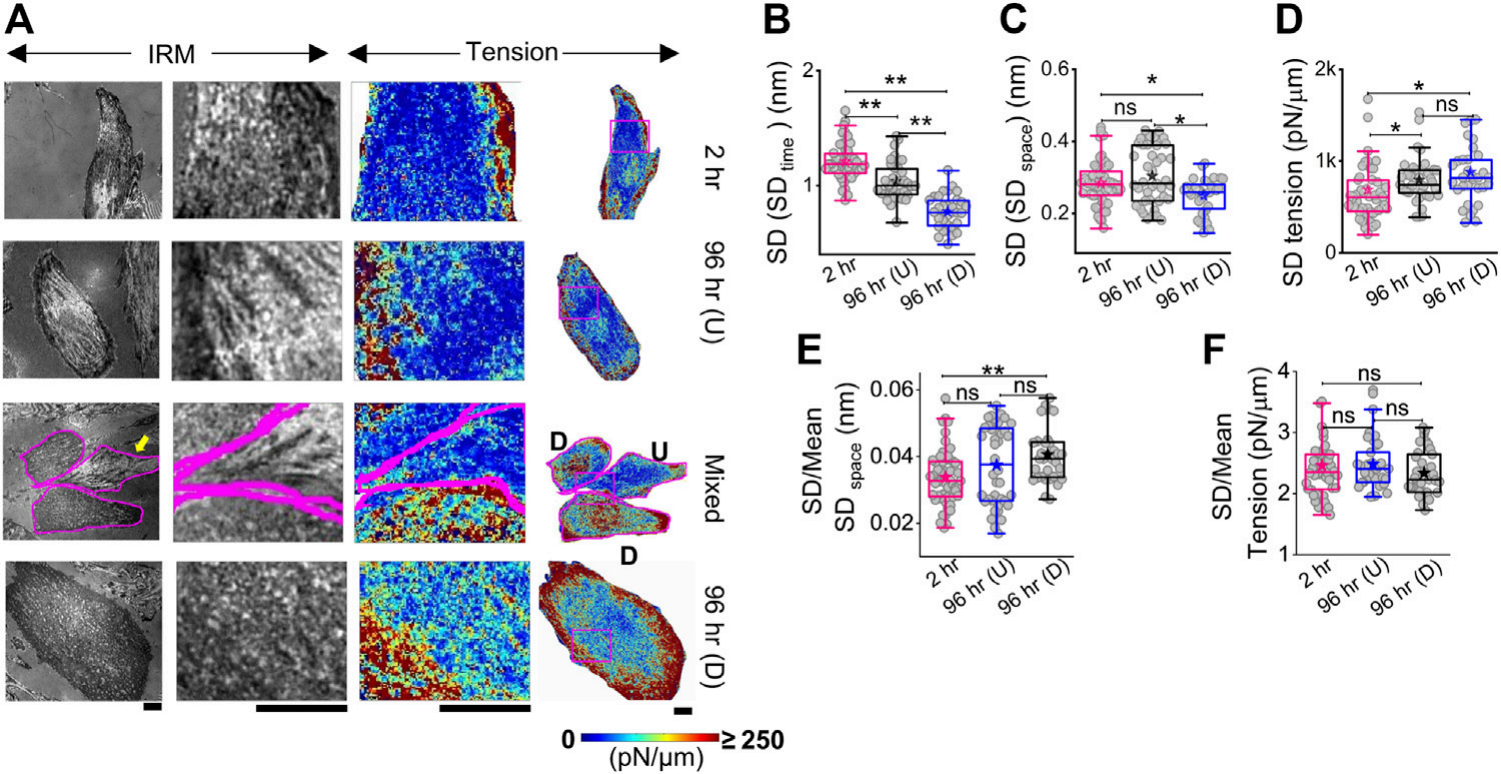
Mapping fluctuation tension to reveal the intracellular heterogeneities. **(A)** Representative IRM images (first column), zoomed-in IRM region (second column), and zoomed-in and overall tension map (third and fourth columns) of cells at 2 h, undifferentiated cells at 96 h, and differentiated cells at 96 h. Regions zoomed in are outlined as pink rectangles in the last column of images. Scale bar = 20 µm for zoomed-out images. D denotes differentiated cells, and U denotes undifferentiated cells in row named “Mixed”. **(B)** Intracellular temporal fluctuation and **(C)** heterogeneity comparison among three populations of cells using FBRs of size 4 × 4 pixels (0.72 × 0.72 µm^2^) for each cell. **(D)** Intracellular membrane tension heterogeneity comparison and **(E,F)** mean-normalized standard deviations (**E**: SD_space_ and **(F)**: tension) among three populations of cells. For B–D: n = 3 independent experiments with 52 cells (2 hr), 40 cells 96 hr (U), and 32 cells 96 hr (D). The Mann–Whitney U statistical significance test is performed; ** denotes *p* value <0.001 and * denotes *p* value <0.05; ns = not significant.

The local tension profile is best assayed by measuring the tension at each pixel and visualizing it as a map (Figure 3A, Supplementary Figure S4 **)**. However, since it included pixels that were not FBRs, it must be noted that the map is mainly for visually assessing the spatial tension distribution. We, therefore, corroborated this data with measurement of the intracellular variation of fluctuations (SD (SD_time_) and SD (SD_space_)—Figures 3B,C; Supplementary Table S1) and tension (SD_tension_—Figure 3D; Supplementary Table S1) measured using FBRs. Maps indicated that at 2 h post administration of DM, tension is high at the cell periphery. A typical image of differentiated (D) cells in close contact with an undifferentiated (U) cell (Figure 3A third row) showed higher tension than the undifferentiated, with edges more adhered and higher in tension. At 96 h, cells revealed higher variability in tension (Figure 3D, Supplementary Figure S4), but intracellular variability in spatial local undulations and temporal fluctuation are either unchanged or reduced. On differentiation, mean tension increased, while the mean of fluctuation amplitude (SD) decreased. To remove the effect of the changing mean, we plotted the mean-normalized standard deviation, SD, using SD/(mean of SD) (Figure 3E) and carried out the same for tension (Figure 3F). The normalized variation increased for SD_space_, whereas for tension, no change was observed. Together, this implied that the enhanced variation of tension from region to region is connected to the increase in mean tension and slightly enhanced intrinsic variability of fluctuations.

Thus, differentiation is demonstrated to affect overall tension and the intracellular variation of membrane fluctuations in cells. To understand how tension might be important in deciding cell fate, we next follow the same cells through differentiation.

### Tension trajectories of single cells are connected to their final differentiation state

**FIGURE 4:**
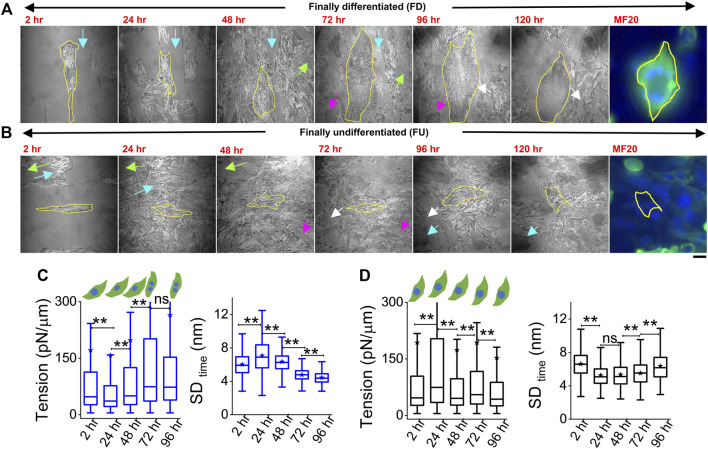
Tracking particular cells through the differentiation period. **(A)** Representative image-frames of a followed cell which finally differentiated (FD), tracked for 5 days of differentiation with its corresponding MF20 (after fixation at 120 h). Same color arrow-pairs on adjacent timepoints point out regions used as references to relocate the cells at the later timepoint. **(B)** A typical cell which does not fuse or remains finally undifferentiated (FU), tracked for 5 days of differentiation with its corresponding MF20 (after fixation at 120 h). **(C)** Membrane tension and SD_time_ comparison of a typical FD cell shown in **(A)** 3,385 (2 h), 6,239 (24 h), 6,798 (48 h), 6,711 (72 h), and 5,089 (96 h) FBRs are used to compare SD_time_; 2,563 (2 h), 2,560 (24 h), 2037 (48 h), 3,053 (72 h), and 2,209 (96 h) FBRs (4 × 4 pixels or 0.29 × 0.29 µm^2^) are used to compare tension. **(D)** Membrane tension and SD_time_ comparison of a typical FU cell shown in B; 3,020 (2 h), 2,638 (24 h), 4,615 (48 h), 6,172 (72 h), and 7,118 (96 h) FBRs are used to compare SD_time_; 1,342 (2 h), 560 (24 h), 2,346 (48 h), 2,750 (72 h), and 2,997 (96 h) FBRs are used to compare tension. Scale bar = 20 µm. The Mann–Whitney U statistical significance test is performed; ** denotes *p* value <0.001; ns = not significant.

Cells were plated on line-shaped micropatterns (Pontes et al., 2017; Kumar et al., 2019) that were 20 μm wide, interspersed with 15-μm-wide low-adhesive, PEG-coated regions. This controlled their initial orientation and lateral spacing, although we also observed heterogeneities and cell growth crossing over patterns. Cells were tracked through 4 days of treatment with DM at 2, 24, 48, 72, and 96 h. They were taken out from the incubator for imaging—only for 30–60 min per day, thus minimizing physiological stress. To find back the same coordinates on the dish each day, the dishes were marked with two reference points whose coordinates were noted. Comparing old and new coordinates of reference points helped in the calculation of transformations necessary (rotation/translation) to find previously imaged regions. Using beads of diameter 2 μm, we estimated an error of 19 ± 10 μm in the prediction of coordinates (Supplementary Figure S5). During experiments, this resolution together with reference regions (Figures 4A,B arrows, Supplementary Figure S5C) was usually enough to locate back cells. Figure 4 shows two typical cells (Figures 4A,B) where the same cell was first imaged at five different timepoints (2 h–96 h). The multinucleated state (clear from DIC images) and distinctive IRM pattern (Figure 1B) were used to demarcate differentiated from undifferentiated cells during live experiments.

However, further validation was also performed in multiple typical experiments by following live cell IRM measurements with cell fixation and subsequently staining cells with MyHC antibody (MF20) at 120 h (last image, Figures 4A,B). Cells identified as differentiated (by IRM morphology and multinucleated state seen in DIC) showed significantly higher intensity (Figure 4A, last image) than the ones identified to be undifferentiated. (Figure 4B, last image). Typical tension trajectories for these cells are shown in Figures 4C,D (Supplementary Table S1).

**FIGURE 5:**
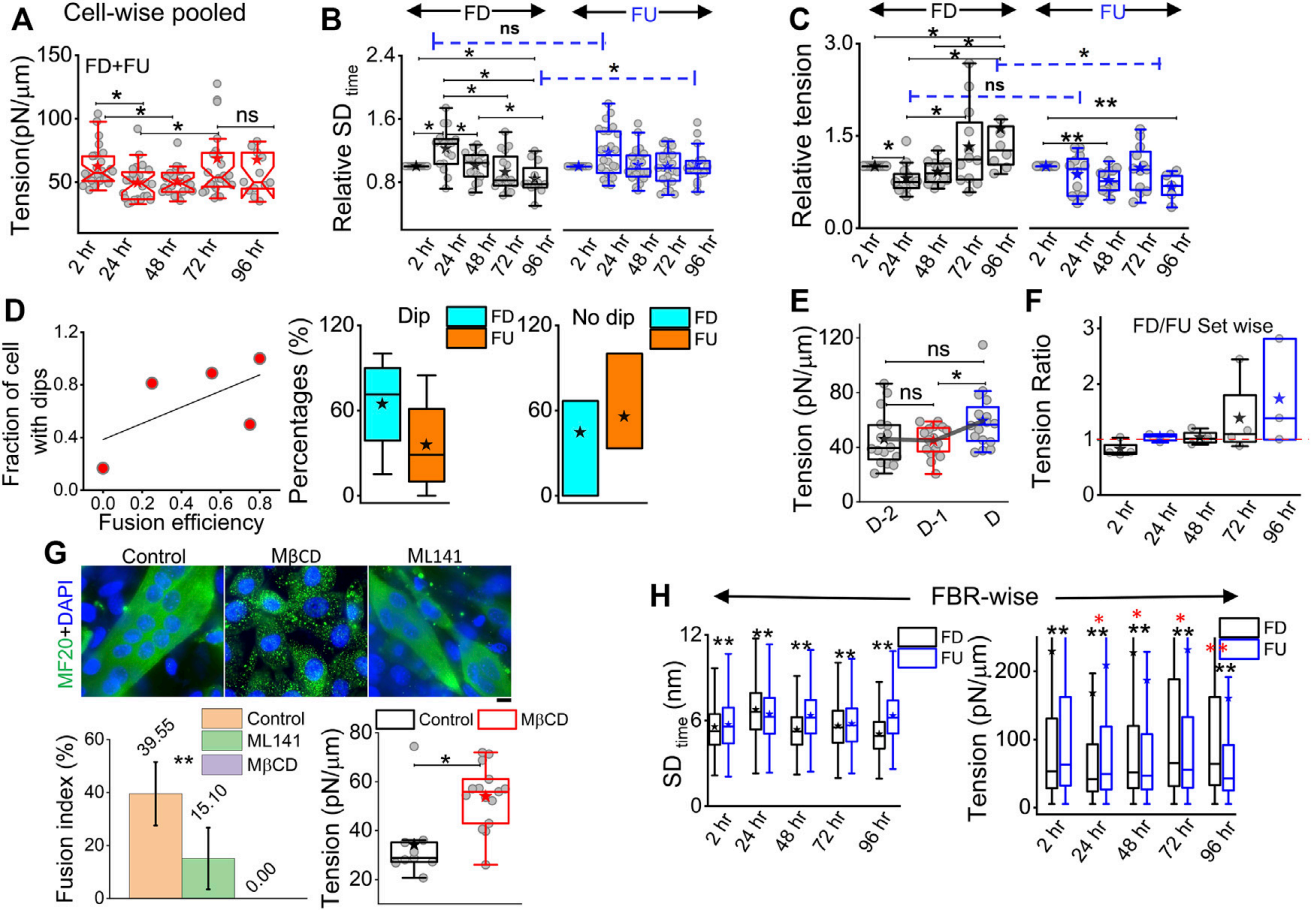
Comparing membrane fluctuation and tension between cells of different final fates. **(A)** Tension of the cells (finally differentiated (FD) and finally undifferentiated (FU) pooled together followed through time. **(B)** Relative comparison of single-cell SD_time_ in FD and FU pools. Values represent fold change with respect to measurement carried out at 2 h for the same cell. **(C)** Relative cell-wise comparison of tension in FD and FU cells. **(D)** Left: visualization of the fraction of cells in a set, which display a transient tension decrease at 24 h and the observed fusion efficiency in that set. Line denotes linear fit. Center: plot of cells that display dips. The percentage of such cells in sets that finally differentiate (FD) or remain single (FU). Right: plot of cells that do not display dips. Percentage of such cells in sets that finally differentiate or not. **(E)** Tension comparison of FD cells pooled with respect to the day of fusion **(D) (F)** Tension ratio change over days, and values represent the ratio between FD and FU population in different sets. **(G)** Immunofluorescence of MF20 and DAPI staining for control, MβCD-treated, and ML141-treated cells on day 5 of exposure to differentiating media. Plot uses 16 myotubes from a single set. Data are representative of two independent experimental sets. The tension plot compares the tension in control and MβCD (2 mM, 24 h)-treated cells. **(H)** Comparison of local (FRB-wise) SDtime and tension between FD and FU pools at different hours after treatment with differentiation media. Unless otherwise stated, data are representative of *n* = 5 independent experimental sets with 12*5 timepoints (FD) and 12*5 timepoints (FU) cells used to obtain 82,893 (2 h), 57,773 (24 h), 85,871 (48 h), 66,018 (72 h), and 91,448 (96 h) FBRs (4 × 4 pixels) for FD cell fluctuation analysis and 87,626, 71,161, 109,153, 91,307, and 28,950 FBRs (4 × 4 pixels = 0.72 µm^2^), respectively, for FU cells’ fluctuations analysis; after fitting 43,800 (2 h), 24,452 (24 h), 22,179 (48 h), 35,653 (72 h), and 40,465 (96 h) FBRs (4 × 4 pixels) used for FD cell tension analysis and 34,726, 25,030, 39,562, 39,409, and 11,450 FBRs (4 × 4 pixels = 0.29 × 0.29 µm^2^), respectively, were used for FU cell tension analysis. The Mann–Whitney U statistical significance test is performed, ** denotes *p* value <0.001, and * denotes *p* value <0.05; ns = not significant. The linear mixed-effect model (LMM) was performed for those FBR-wise comparisons for which the *s are represented in red.

On pooling all cells irrespective of their differentiation status, we found that tension showed a slow reduction that was recovered back (Figure 5A). To understand the relative tension change experienced by single cells, each cell’s SD_time_ or tension at 2 h was used to normalize its tension measured at other timepoints (Figures 5B,C; Supplementary Table S1). Cells that finally differentiate (FD) were found to be more prone to having a temporary increase in fluctuations (Figure 5B) or decrease in tension (Figure 5C) at 24 h than cells that remained finally undifferentiated (FU) till 96 h. The transient increase in SD_time_ (Figure 5B) was found to be a significant parameter demarcating trajectories of FD and FU cells. The decrease in tension (or increase in fluctuations) was usually (for 80% cells) followed by a recovery at 48 h. We term them as “dips” and find for each set how the percentage of cells with dips couple to the fusion efficiency (number of cells that fuse/total cells observed) (Figure 5D left). Were cells with dips more prone to a particular fate? We did not find compelling evidence, but the data (Figure 5D center, right) suggested that among cells with dips, a higher percentage fused, whereas among cells without dips, a higher percentage remained single. However, mean tension arranged with respect to the day of fusion (D) showed no particular pre-tension drop (Figure 5E). When the mean tension of FD cells was compared to that of FU cells, the ratio was found to be initially lower than 1 followed by an increase (Figure 5F). Before further exploring this, we demonstrated the effect of increasing tension externally by continuous cholesterol depletion using MβCD (Zidovetzki and Levitan, 2007). A milder concentration (2 mM) was chosen at which the cellular morphology, spreading, and packing remain unaffected (Figure 5G top panel). We found that at this concentration, MβCD increased tension and cells completely failed to fuse even after 5 days of exposure to differentiation media (Figure 5G; bottom panel). Was it due to disturbed tension drop or tension regulation or caused by other effects of cholesterol depletion? Although we hope to address those in the future, it must be noted that the cell–cell distances were not affected by this treatment.

To further evaluate the role of tension, we next studied the effect of a well-known tension-regulating endocytotic pathway, CG pathway, by ML141, a known inhibitor of CDC42 GTPase (Thottacherry et al., 2018). We observed a significantly reduced fusion index (Figure 5G), whereas our research showed that ML141 action increased the tension of C2C12 cells (Supplementary Figure S6E). We, thus, showed that overall tension regulation is essential for fusion. However, at the single-cell level, does tension control its ability to fuse? We compared the tension measured in cells much before the cells finally fuse (FD) or fail to fuse (FU) and found that at the 2-h timepoint, cells that finally fuse had smaller median tension than cells that fail to fuse; however, the differences were statistically not significant when also checked using LMM (Figure 5H, Supplementary Figure S6). At 24 h, the 2-hr trend was found to be reinforced for tension, making the change significant now and supporting the need for an early low-tension state for successful fusion. SD_time_ also showed softening at 24 h (Figure 5H left). A reversal occurred post 48 h when FD cells were more tensed with lesser fluctuations—probably due to progress into differentiation. However, the hypothesis that a tension dip is required for successful fusion later would predict a decrease of the FD/FU tension ratio from 2h to 24 h, having different tensions to start with, which would obscure the predicted effect. An early role of regulation of fluctuations and tension in determining cell fate was, hence, implicated by these findings.

Since myomerger has been ascribed the role of stressing hemi-fused membrane, we next explored its interplay with tension at the population level and its coupling to tension in single cells at different phases of differentiation. We first quantify changes in the surface level of myomerger with the progress of differentiation.

### Distribution of surface myomerger

**FIGURE 6:**
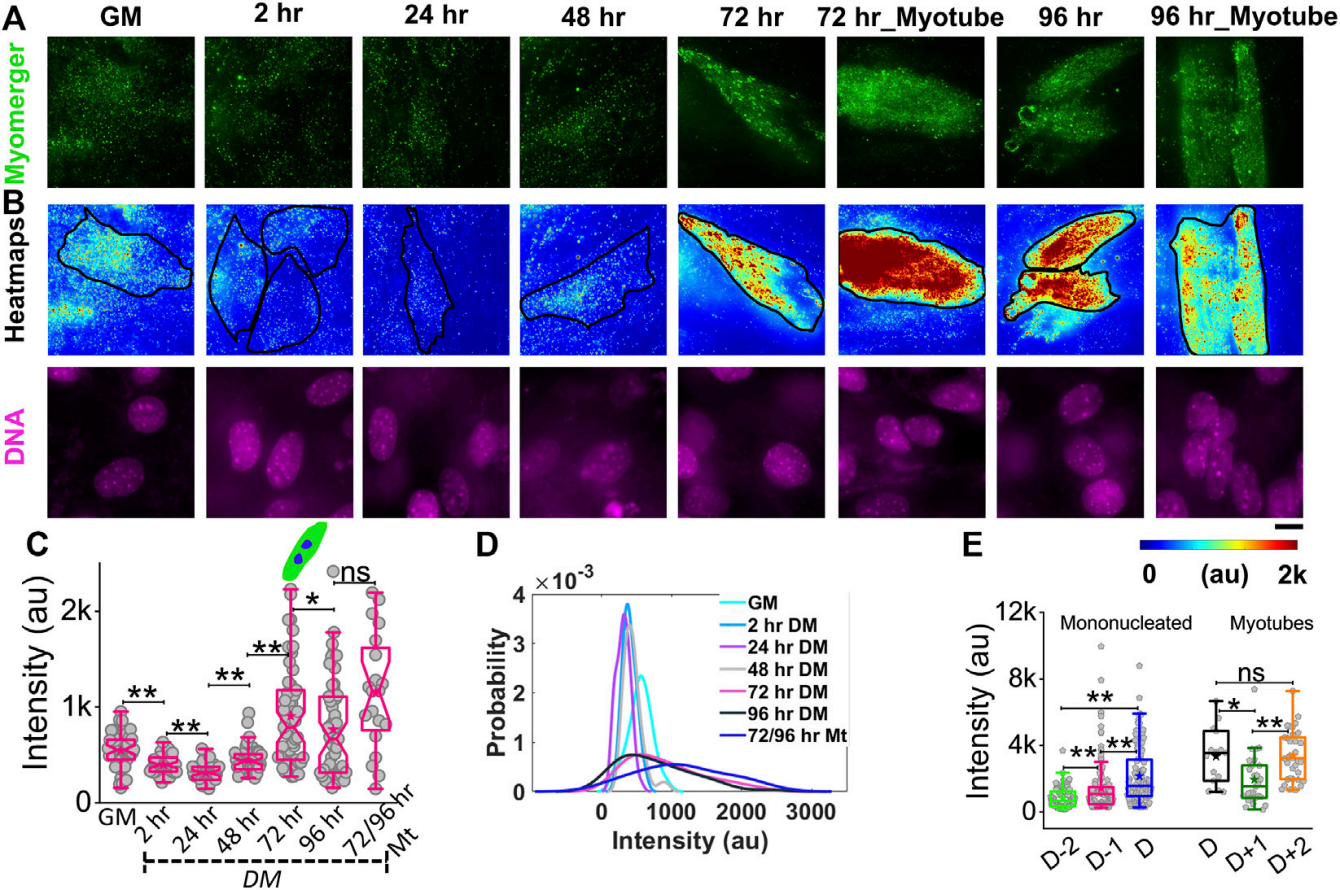
Phenotypic distribution of the surface myomerger during differentiation. **(A)** TIRF images of myomerger immunofluorescence of a representative differentiating set. Cells were fixed at different timepoints but imaged together. **(B)** Corresponding color-coded intensity images (heat map) and DAPI staining (DNA) included. **(C)** Box plot comparison of the whole-cell averages of myomerger intensity between different timepoints from a single representative set using 52 cells (GM = growth medium), 52 cells (24 h), 51 cells (48 h), 53 cells (72 h), 51 cells (96 h), and 20 cells (72/96 mt: myotubes from 72 to 96 h); *n* = 3 independent repeats.**(D)** Probability density estimates of the mean intensity per cell for each condition for data in C. **(E)** Myomerger intensity compared fusion day wise (D = day of fusion) using cells; *n* = 3 independent experiments with 134 cells (D-2), 137 cells (D-1), and 130 cells **(D)** used for mononucleated cell analysis, and 15 cells (D), 27 cells (D+1), and 34 cells (D+2) are used for myotube analysis. Scale bar = 10 µm. The Mann–Whitney U statistical significance test is performed, ** denotes *p* value <0.001, and * denotes *p* value < 0.05; ns = not significant.

While myomerger abundance is known to be increased with progress in differentiation (Bi et al., 2017), to understand its surface levels and coupling to membrane fluctuations and tension, we use immunofluorescence combined with TIRF microscopy to quantify the endogenous level of the myomerger at the cell surface of single cells (Figure 6, Supplementary Figure S7A). Cells in growth media (GM) have an appreciable level of the surface myomerger, which first could reduce (Figures 6A–C; Supplementary Table S1) and then increase. The cell–cell variability in myomerger levels also increased drastically with time (Figure 6D). We also found sample-to-sample variability in the timing of the initial decrease. Different experiment sets show the transient decrease at either 2 h or 24 h. However, when data from four different sets are collated relative to the day myotube formation starts in the sample (D), the increase was found to start 48 h before D in the dish (Figure 6E, Supplementary Figure S7B). Even within myotubes, the variability was high, and variation with time was observed (Figures 6C–E). It should be noted that the presence of surface myomerger in cells in GM could be substantiated. First, it was found to be more than the non-specific binding of secondary antibodies (Supplementary Figures S7D,E). Next, it could be also measured by checking protein abundance by Western blotting (Supplementary Figure S7F). The fact that Western blot shows a much higher change in myomerger levels at 24 h (in comparison to GM) while changes are less pronounced for surface levels could be because the myomerger is known to be regulated strictly at the membrane, irrespective of its whole cell expression/overexpression status (Golani et al., 2021).

Having established that myomerger shows modulations in its surface levels, in the next part, we check if tension alterations and myomerger alterations are coupled.

### Myomerger expression and its correlation with the spatial tension profile

**FIGURE 7:**
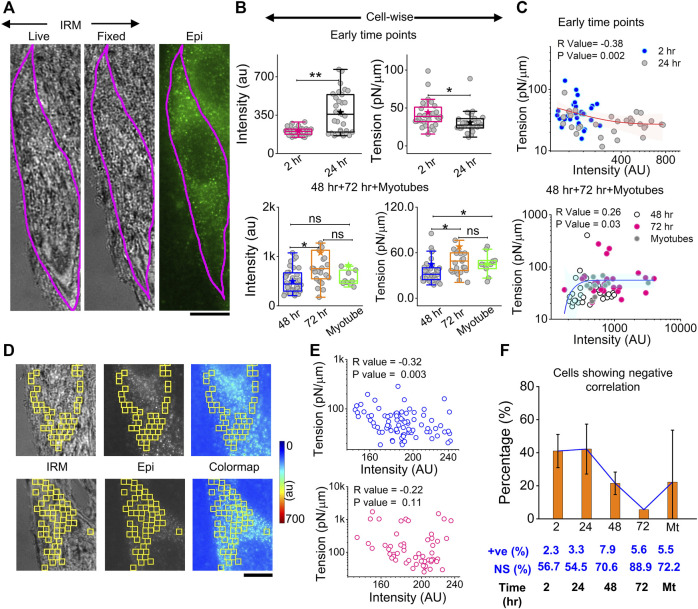
Correlation between myomerger immunofluorescence and fluctuation tension. **(A)** Representative images of a particular cell tracked live in IRM, fixed, and imaged in IRM and epifluorescence (90x) to measure fluctuations, tension, and myomerger immunofluorescence [**(B)**, top panel]. Intensity and tension comparison between 2 h and 24 h; *n* = 3 independent experiments with 32 cells (2 h) and 31 cells (24 h) used for analysis [**(B)**, bottom panel]. Intensity and tension comparison between 48 h, 72 h, and myotubes; n = 3 independent experiments with 38 cells (48 h), 25 cells (72 h), and 10 cells (myotubes) used for analysis. **(C)** Scatter plot to show the correlation between tension and intensity pooled from all early timepoints (2 h and 24 h) (top) and later timepoints (bottom); number of independent tries and cells are same as mentioned in B. Red and blue lines with pink and cyan shades are exponential fits and confidence backs to serve as guides to the eye. **(D)** Representative IRM live, epifluorescence. and color-coded intensity images of a particular cell section—showing analyzed 24 × 24 pixels (1.73 × 1.73 μm^2^) FBR regions in yellow. **(E)** Corresponding scatter plot is shown in the right-top panel, with a significant negative correlation evident from the respective R (Spearman’s correlation test) and *p* values. A scatter plot for a typical cell section with non-significant correlation is shown in the right-bottom panel. **(F)** Percentage of cells displaying negative correlation of local tension and local myomerger. The percentages of positive (+ve) and NS correlation are mentioned below. Scale bar = 10 µm. The Mann–Whitney U statistical significance test is performed, ** denotes *p* value <0.001, and * denotes *p* value <0.05; ns = not significant.

The correlation of local myomerger intensity at the cell surface with its membrane mechanical properties was explored by performing in tandem, live-cell, fixed-cell IRM, and myomerger immunofluorescence (Figure 7A). A delay of ∼15–25 min with respect to the IRM should be noted. Despite the delay, while local trends were expected to change in this timescale, the overall cell intensity, known to change slowly (Figure 6), was expected to remain similar. The immunofluorescence was assayed by regular epifluorescence instead of TIRF after cells are fixed. Parallel checks with the same samples imaged in TIRF and epifluorescence (epi) showed that the trend observed in TIRF was also replicated in epifluorescence (Supplementary Figure S7B).

At early timepoints, cell averages of myomerger intensity increased from 2 to 24 h, while the tension in this pool decreased (Figure 7B, top panel, Supplementary Table S1). The membrane confinement parameter and effective cytoplasmic viscosity also decreased (Supplementary Figure S8A). At later timepoints, intensity increases from 48 to 72 h, but in myotubes, the increase was not appreciable. Tension also increased in single undifferentiated cells from 48 to 72 h, with the increase in myotubes not significant (Figure 7B, bottom panel, Supplementary Table S1).

To further corroborate, instead of comparing pools, cell averages of intensity and tension were correlated cell-wise, and a significant negative correlation at early timepoints (and lower myomerger intensity) was noted (Figure 7C, top). The correlation turned positive at later timepoints and for myotubes (all higher intensities) (Figure 7C, bottom). It is, thus, clear that the overall myomerger intensity has a biphasic relationship with cell tension.

To address if such a coupling exists at a local length scale, we aligned images to make the best possible matches of the live and fixed-IRM images and used common regions (between myomerger fluorescence and FBRs of live IRM) that were 24 × 24 pixels or 1.73 µm × 1.73 µm wide (Figure 7D images). Typical data (from particular cell sections) of measured tension in each region vs. the measured mean intensity at the same FBR could display a non-significant correlation (Figure 7E, lower row) or a negative correlation (Figure 7E, upper row). Like tension, SD_time_ and SD_space_ also showed a significant correlation (positive for most early cells, Supplementary Figure S8B). On collating cell sections from three sets, ∼40% of samples of early timepoints showed a negative correlation that reduced drastically at later timepoints (Figure 7F). Hence, we found the negative correlation to be prominent at early timepoints even on comparing single-cell intracellular regions—strongly indicating that myomerger locally reduces tension. The positive correlation of cellular myomerger was not locally evident within the ∼1.73-µm length scale.

We next quantified how myomerger’s intensity distribution changed as its correlation with tension was lost—either at later timepoints and myotubes or by cholesterol depletion at early timepoints. We checked if there were any common features.

### Diffused myomerger distribution in myotubes and cholesterol depletion

**FIGURE 8:**
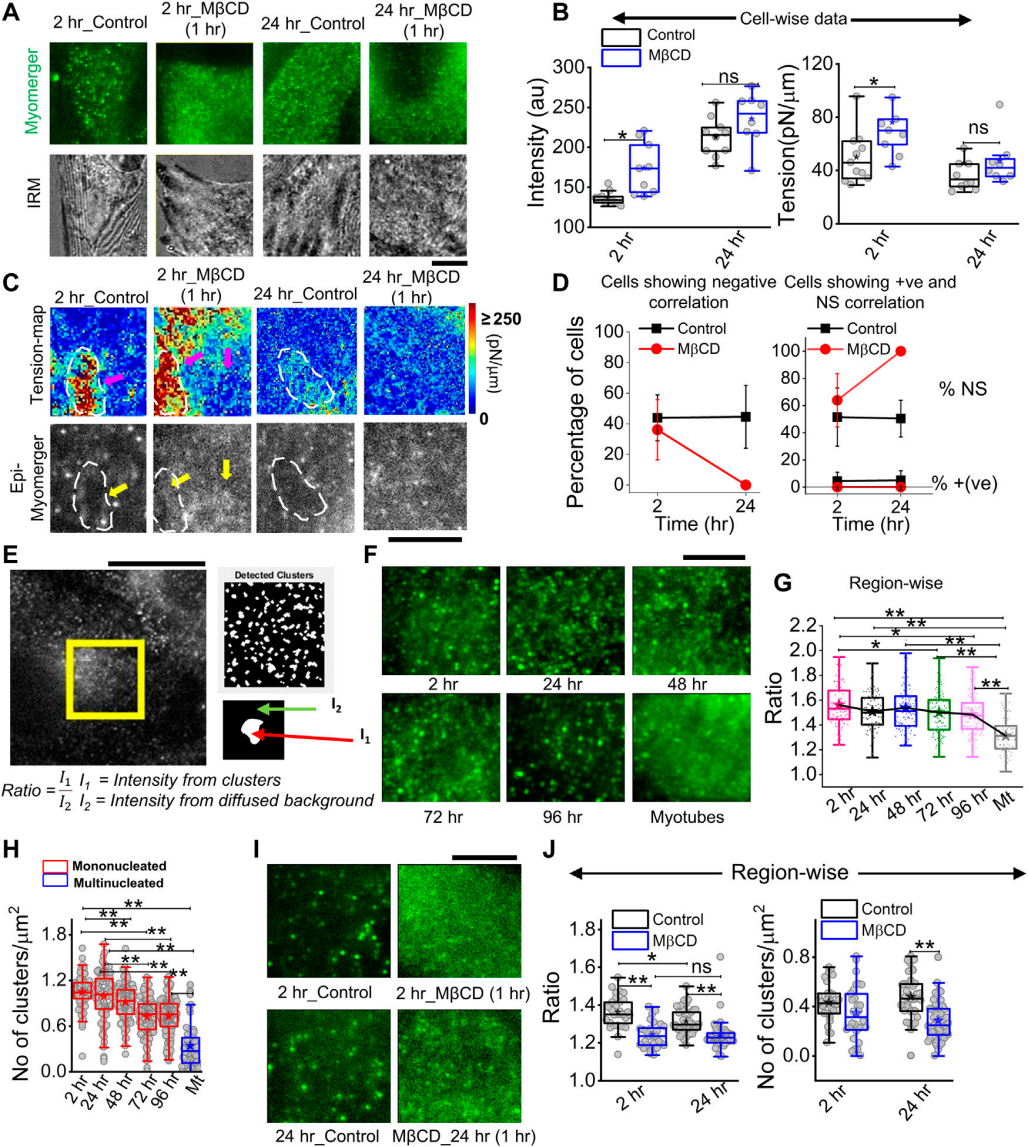
Cholesterol dependence of myomerger surface distribution. **(A)** Myomerger immunofluorescence of 2 h and 24 h DM-treated cells in control and MβCD-treated (10mM, 1 h) conditions. The bottom row shows IRM (live) of the same regions of respective cells prior to fixation. **(B)** Cell-wise comparison of myomerger intensity and tension in control and MβCD-treated conditions for 2 h and 24 h DM-treated cells. **(C)** Tension maps of cell-regions mentioned in (A) and corresponding epifluorescence images (bottom). White dashed outlines and arrows are guides to the eye to compare fluorescence at those regions with the corresponding tension. **(D)** Percentage of cells with negative correlation (left) between myomerger intensity and tension reduces on MβCD treatment. Percentage of cells showing positive correlation or ns also plotted (right). **(E)** Representative images of myomerger immunofluorescence and (left) detection of clusters and (bottom) use of white and black regions of the binary image as masks for getting mean intensity of pixels (of original image) in clusters (I1) and those on the background (I2). **(F)** Zoomed-in view of myomerger immunofluorescence displaying qualitatively changing sharpness of the clusters with time after administering differentiation media. **(G)** Quantification of the ratio of mean intensities inside clusters and the mean background diffused intensity. A decreasing trend is observed. **(H)** Decrease in the number of punctas with days. **(I)** Zoomed-in view of myomerger immunofluorescence after cholesterol depletion by MβCD (for 2 h). **(J)** Cholesterol depletion decreases the ratio for cells exposed to 2 h or 24 h of differentiation media (left). Decrease in the number of punctas after cholesterol depletion (right). Scale bar = 5 µm. The Mann–Whitney U statistical significance test is performed, ** denotes *p* value <0.001, and * denotes *p* value <0.05; ns = not significant.

Immunofluorescence (IF) images taken in epifluorescence (followed by IRM) helped visualize a punctate distribution at 2 h and 24 h that drastically affected by cholesterol depletion (Figure 8A). The mean intensity increased on cholesterol depletion, while tension also increased—an effect more pronounced for cells at 2 h than 24 h post-trigger (Figure 8B). Although a perfect matching between fluorescence and tension is not possible, we next tried to visualize the correlation (and its loss) inside ∼ 8 × 8 μm^2^ regions (Figure 8C). We observed a lower myomerger intensity in regions where the tension was locally high (white outlines, Figure 8C). However, it should be noted that after cholesterol depletion, the diffused fluorescence was hardly anti-correlated with any tension heterogeneity (Figure 8C). The percentage of cells with negative correlation reduced on MβCD treatment at 2 h but are not found at all at 24-h’s cholesterol depletion (Figure 8D). The strength of the correlation coefficient was also reduced (Supplementary Figure S9). On the one hand, this implied increasing tension does not decrease myomerger intensity, and on the other hand, the punctated nature of the myomerger is suggested to be important for the negative correlation. Were punctas or clusters really lowered on cholesterol depletion? And, did it also happen when at later timepoints, the negative correlation is lost? We detected the clusters by object detection in MATLAB and found the ratio of mean intensity in clusters to that of the diffused background (Figure 8E). We first showed that the ratio decreased as cells remained in differentiation media for a long time (Figures 8F,G) and the number of clusters decreased (Figure 8H). Analysis of the zoomed-in view of myomerger IF (Figure 8I) showed that the ratio and number of punctas per unit area also reduced on cholesterol depletion (Figure 8J). Thus, the negative correlation was strongly dependent on the myomerger’s presence as clear punctas/clusters.

## Discussion

 In this study, the use of IRM enabled a non-invasive measurement of membrane mechanics of the differentiating C2C12 cells even at higher cell densities. Although IRM limits measurements to the basal membrane, which does not take part in the fusion process, it helped in recording the global mechanical changes triggered by differentiation. We found that cell–cell fusion resulted in enhanced cell surface tension (Figures 2G, 5C), which is in line with higher cortical tension reported in insect myotubes (Kim et al., 2015). We, too, observed that myotubes had a thicker actomyosin cortex (Supplementary Figure S2B), and a higher effective viscosity (η_eff_, Supplementary Figure S3D) is experienced by the membrane while fluctuating. We also showed here that myotubes [96 h (D), Figure 5C] have higher fluctuation–tension than the cells they originate from, unlike mono-nucleated cells at the same timepoint [96 h (U), Figure 5C]. While the effect of differentiation on tension was thereby clear, the role of tension in cell fate determination during fusion could be derived mainly from single-cell tracking experiments.

Stochasticity in cell fate determination has been observed in many systems (Johnston and Desplan, 2010), including cell–cell fusion (Capkovic et al., 2008; Trapnell et al., 2014; Arnold et al., 2020). Cells that fail to fuse in a given time window, however, have been shown to retain fusion potential (when triggered again afresh) (Yoshida et al., 1998). Despite cell-to-cell variability, our measurements indicate that cells that reduce tension upon being triggered are more probable to finally differentiate (FD, Figure 5). A higher percentage of FD cells (than FU cells) were found to have displayed a dipping tension at 2 h or 24 h post DM. Why the dip is needed is unclear, but the functional significance of early tension regulation is further supported by the observation of loss of fusion by enhancing tension throughout using cholesterol depletion (by MβCD). Past studies by other groups administering MβCD in shorter time windows at later timepoints (24 h and beyond and followed by washes) do not report such loss of fusion (Mermelstein et al., 2004). Our checks under such conditions also show no loss of fusion when MβCD was washed away after 30 min (Supplementary Figure S10). Hence, we establish the role of continuous tension regulation in the early phase.

At this point, a potential direct role of cholesterol in fusion or its effect by disrupting caveolae (Dewulf et al., 2019) cannot be ruled out. However, studies on caveolae-forming CAV3 have reported conflicting roles. CAV3 can both help in disrupting the fusion of myoblasts (Volonte et al., 2003) and aiding fusion (Galbiati et al., 1999; Stoppani et al., 2011). Our observation of fusion-defect using ML141, and its tension-enhancing effect on C2C12 (Supplementary Figure S6E), further establishes the central role of tension regulation.

The role of the myomerger that we propose in this early regulation is new. Myomerger has been shown to be important only after hemi-fusion. However, our data capture myomerger’s biphasic, concentration-dependent correlation with tension, suggesting a strong connection between its surface levels and the early stages of differentiation. Despite reports of a monotonic increase in the myomerger’s protein abundance (comparing Western blots using the same protein mass) with the progress of differentiation (Bi et al., 2017), we must note that studies have also shown independent regulation of surface levels (Golani et al., 2021). The increasing phase of myomerger starting ∼48 h before fusion (Figure 6E) was the most robust and relevant observation. In this phase, a substantial negative correlation of myomerger intensity and tension was observed at multiple levels, implying strongly an early role of the myomerger. At and beyond 24 h, the correlation of higher intensities at lower-tension regions was visually clear from cell averages (Figures 7A–C), within single cells (Figures 7D–F), and inside ∼8 μm × 8 μm regions (Figure 8C). The interaction of the ectopic domain of the protein with the membrane (mimicking protein adsorption) and inducing a positive spontaneous curvature could be responsible. Protein adsorption captured as a difference in spontaneous curvature under the protein patch and the surrounding has been implicated to result in local and overall tension decrease (Rangamani et al., 2014). Our observation of the strongest correlation and the highest cluster-to-diffused-background ratio of the myomerger at 2 h (Figures 8G,J) supports the possibility. On cholesterol depletion, the myomerger surface levels enhanced—without substantial change in whole cell myomerger levels (Supplementary Figures S9D,E)—the ratio decreased while the negative correlation was also lost/reduced. Such an early loss of negative correlation (by MβCD) was observed to be detrimental for fusion. High myomerger levels before the onset of hemi-fusion have been reported to reduce fusion (Golani et al., 2021).

At ≥ 48 h, myomerger’s surface level increased and its cell averages correlated positively to tension, but only a very small percentage of region-wise correlations actually was significantly positive (Figure 7F). Although unclear at this stage, it is in line with its indirect effect in stressing distal membranes through its induction of positive curvature on proximal membranes (Golani et al., 2021).

As limitations, we cannot negate the fact that these are clusters of immunofluorescence (IF). Although they reflect a local increase in protein density, the effect of the antibody’s interaction in their formation warrants consideration. However, since the clustering was coupled to the tension measured at the live state, we believe the changes in distribution (clustered to diffused) were faithfully captured by IF. We also note that myomaker’s role in tension regulation should be addressed since it is critical for hemi-fusion. However, its intracellular C-terminal region, which has been shown to be necessary for fusion (Millay et al., 2016), implicates a signaling-based role more than its function as a tension regulator.

In summary, we show early tension lowering is critical for C2C12 differentiation and myomerger plays an active role in this, possibly by its ability to induce local positive curvature. MβCD-based cholesterol depletion obstructs this by reducing local contrast in myomerger’s concentration, enhancing surface and decreasing its tension-reducing ability—thereby suppressing fusion. MβCD’s use in therapeutics makes these observations relevant for future investigations on its effect on muscle health since the fusion of satellite cells to existing myofibers may involve similar tension regulation requirements.

## Star methods

### Key resources table

**Table:** 

Reagent or resource	Source	Identifier	
**Antibodies**			
Myosin 4 monoclonal antibody (MF20)	eBioscience	Cat# 14-650–82; LOT# 4301341; RRID# AB_2572893	
ESGP polyclonal antibody	Invitrogen	Cat# PA5-47639; LOT# UJ2868751A; RRID# AB_2610690	
Goat anti-mouse IgG H&L, Alexa Fluor 488	Abcam	Cat# ab150117; LOT# GR3192504-1; RRID# AB_2688012	
Donkey anti-sheep IgG (H + L) secondary antibody, Alexa Fluor 488	Invitrogen	Cat# A11015; LOT# 2079356; RRID# AB_2534082	
**Cell culture and IF chemicals**			
Cell line (C2C12)	ATCC	Cat# CRL-1772; RRID# CVCL_0188	
Dulbecco’s modified Eagle’s medium (DMEM, high glucose)	Gibco	Cat# 11965092	
Fetal bovine serum, certified, heat inactivated, US	Gibco	Cat# 10082147	
Antibiotic–antimycotic (100X)	Gibco	Cat# 15240062	
Horse serum, New Zealand	Gibco	Cat# 16050122	
Insulin solution human	Sigma-Aldrich	Cat# I9278; LOT# SLBX0973	
Trypsin-EDTA (0.25%), phenol red	Gibco	Cat# 25200072	
Paraformaldehyde	Sigma-Aldrich	Cat# P6148	
Phosphate-buffered saline	Sigma-Aldrich	Cat# P3813	
Gelatin	Sigma-Aldrich	Cat# G2500; LOT# SLBX2973	
Glycine	Merck	Cat# 56-40-6	
Triton X-100	Sigma-Aldrich	Cat# 101371902	
DAPI	Sigma-Aldrich	Cat# D9542; Batch# 0000081272	
PLL-g-PEG	SuSos	Cat# (20)- [3.3]- (2)/3.6 51% LOT# SZ38-35	
Methyl-β-cyclodextrin	Sigma-Aldrich	Cat# 332615-5G; LOT# STBH9769	
ML141	Sigma-Aldrich	Cat# SML0407-5 MG; Batch# 0000067576	
**For calibration**			
NIST traceable particle size standard, 60 µm	Bangs laboratories	Cat# L130806L; LOT# 11,247	
FluoSpheres Size Kit #1, red fluorescent (580/605), 2 µm	Invitrogen	Cat# 1971894	
**Software and algorithms**
ImageJ (FIJI)	NIH	N/A	
Origin	OriginLab Corporation	N/A	
MATLAB	The Mathworks, Inc	N/A	

## Resource availability

### Lead contact

Further information and request for resources and reagents should be directed to and will be fulfilled by the lead contact, Bidisha Sinha (bidisha.sinha@iiserkol.ac.in).

### Materials availability

All new materials and methods generated in this study will be available upon request from Bidisha Sinha (bidisha.sinha@iiserkol.ac.in).

### Data and code availability

Data and custom codes used in this study for analysis will be available upon request to the lead contact.

## Experimental model and subject details

### Cell line

The mouse myoblast cell line from ATCC (CRL-1772) was used as a model system to study fusion and differentiation.

## Method details

### Cell culture and immunofluorescence

C2C12 cells were grown in growth media composed of Dulbecco’s modified Eagle’s Medium (DMEM, Gibco, Life Technologies, United States), 10% fetal bovine serum (FBS, Gibco, HI, US origin), and 1% antibiotic–antimycotic solution(Gibco). For differentiation, cells were seeded at a density of ∼50,000 cells/ml in customized round glass-bottom dishes and maintained in growth media. On attaining 60% confluency, the medium was replaced with differentiation media (DM) consisting of DMEM, 2% horse serum (HS) (Gibco), 1% antibiotic–antimycotic solution, and 0.1% insulin (1 μM) (Sigma-Aldrich) solution. Cells were maintained at 37°C in a humidified incubator with 5% CO_2_ for 5 days in DM, and the medium was replaced every 24 h. All cells had been used within passage number 12. For immunofluorescence, cells were fixed with 4% paraformaldehyde (Sigma-Aldrich) for 15 min, washed properly with phosphate-buffered saline (PBS, Sigma-Aldrich), incubated in 0.1 M glycine (Sigma-Aldrich) for 5 min, and then washed again in PBS. For blocking, cells were incubated with 3 ml of 0.2% gelatin (Sigma-Aldrich) solution for 3 h at room temperature. Primary antibody treatment was carried out with ESGP polyclonal antibody (Invitrogen, PA5-47639) at 1:200 dilution in a blocking agent and kept at 4°C overnight. Donkey anti-sheep Alexa Fluor 488 secondary antibody (Invitrogen, A-11015) was used after washing the cells with PBS and kept for 2 h. DAPI (Sigma-Aldrich) with 1:1,000 dilution was used for nucleus visualization. Cells were washed after each step with PBS, and fixed cell imaging was carried out finally in 2 ml of PBS. Cells were checked for *Mycoplasma* and tested negative. Institutional Biosafety Committee approved the use of C2C12 cells.

### Drug treatment

Differentiated cells were treated with methyl-β-cyclodextrin (MβCD) (Sigma-Aldrich) and ML141 (Sigma-Aldrich) at different timepoints of differentiation. To check the effects of these two drugs on fusion efficiency separately, MβCD (2 mM) and ML141 (5 µM) were added directly to differentiation media from the beginning of the differentiation and followed up to 96 h. For the correlation studies, a higher concentration of MβCD (10 mM) was used, and treatments were carried out at separate timepoints with 1 h incubation time with the drug.

### Micropattern protocol

Etched glass coverslips (1:19 acetic acid and ethanol solution) were used; first, we cleaned it using a UV/–Ozone (UVO) cleaner (Jelight Company, United States) for 5 min. Cleaned coverslips were incubated with 0.2 mg/ml PLL-g_PEG (SuSos, Switzerland) for 2 h. A cleaned photomask (JD Photo Data, UK) was used to pattern on PEG-coated coverslips. The line-2 (width:20 μm; length: no constraint; and spacing: 15 µm spacing) pattern was used. The coated coverslips were placed on top of the pattern of interest on the photomask and a small drop of water was sandwiched in between. Patterning was carried out inside a UVO cleaner for 5 min. Patterned coverslips were removed from the photomask by floating them off with water. Cells were seeded on the patterned side (Kumar et al., 2019).

### Interference reflection microscopy

For interference reflection microscopy (IRM), we used an Eclipse Ti-E motorized inverted microscope (Nikon, Tokyo, Japan) equipped with adjustable field and aperture diaphragms, 60x Plan Apo (NA 1.22, water immersion) with ×1.5 external magnification. The EMCCD camera (Evolve 512 Delta, Photometrics, United States) and CMOS (ORCA Flash 4.0 Hamamatsu, Japan) camera were used for IRM, epifluorescence, and DIC modes of imaging. A 100-W mercury arc lamp and a 546 ± 12 nm interference filter with a 50:50 beam splitter were additional requirements for IRM mode. All cells were imaged in 3 ml of DM, and calibration was carried out using 60 µm diameter polystyrene NIST beads (Bangs Laboratories Inc.). Fast time-lapse images of cells were taken at 20 frames per second (50 ms interval) in the CMOS camera or 19.91 frames per second in the EMCCD camera. In total, 2048 frames were captured for IRM analysis.

### Total internal reflection fluorescence microscopy and epifluorescence microscopy

For TIRF, an Olympus IX-83 inverted microscope (Olympus, Meliville, NY) equipped with a 100 × 1.49 NA oil immersion TIRF objective (PlanApo, Olympus) was used. Images were acquired using a CMOS camera (ORCA Flash 4.0 Hamamatsu, Japan). A 488-nm laser beam was used as a laser source for TIRF. All images were taken at 200 ms exposure time and ∼100 nm penetration depth. For epifluorescence, a mercury arc lamp was used, and images were taken using FITC and DAPI filters with ×60 and ×100 objectives. All images were taken at 200 ms of exposure time.

## Quantification and statistical analysis

### Measuring the fusion index

MF20- and DAPI-labeled myotubes were considered for fusion index calculations. The fusion index was calculated by counting the number of nuclei in a single myotube divided by the total number of nuclei in the same frame, multiplied by 100 for obtaining the percentage.

### Intensity measurement for checking the MF20 level

For measuring intensity, 24 × 24 pixel rectangular regions were selected from each MF20-labeled cell. Mean intensity from all the ROIs is then selected cell-wise to compare.

### Analysis of membrane height fluctuations

This involves, first, a careful calibration of the height–intensity relationship using 60-μm beads for a range of net intensities. Minimum and maximum intensities observed in single cells were then used to select the corresponding calibration curve from the bead data. Next, images of cells were scrutinized pixel by pixel using MATLAB to eliminate pixels that do not lie in the first branch of the intensity–height relationship (within ∼100 nm of the coverslip). This step was performed because the monotonously increasing relation between IRM intensity and membrane height (from coverslip) exists only for the first branch region. Once relevant pixels were identified, different 12 × 12 pixel regions (unless otherwise indicated) corresponding to 2.16 µm × 2.16 µm (or as indicated in figures) were marked out and used to build single-cell statistics. The amplitude of membrane height fluctuations is quantified by the standard deviation (SD) calculated from the height time series of single pixels and then averaged over single FBRs. This parameter is termed SD_time_. The amplitude of spatial undulations was quantified by measuring the SD—but over 12 × 12 pixel regions of single-time-point images. The average of this over 20 frames is used as the SD_space_. To understand the underlying mechanical state, the frequency distribution of the height fluctuations was calculated in the form of the power spectral density (PSD). The PSD was used to calculate from height time-series of every pixel of FBRs either by using the FFT-based method (Biswas et al., 2017; Biswas et al., 2019) or the covariance method (autoregressive PSD estimation) (MATLAB). “Exponent” captures the state of confinement from the power relationship between PSD and frequency in the linear part of the PSD (frequency ranging from 0 Hz to 10 Hz). The PSD was fitted with the Helfrich-based theoretical model $PSD\left(f\right)=\frac{4\eta_{eff}Ak_{B}T}{\pi }\int_{q_{\min}}^{q_{\max}}\frac{dq}{{\left(4\eta_{eff}\left(2\pi f\right)\right)}^{2}+{\left[\kappa q^{3}+\sigma q+\frac{\gamma }{q}\right]}^{2}}$ (Biswas et al., 2017) to estimate the fluctuation-tension (σ), effective viscosity (η_eff_), level of confinement (γ), and the effective active temperature (*A*). The bending rigidity (κ) was fixed at 15 k_B_T. Due to the increasing heterogeneity in the biochemical nature of cells in a population, calibrations were specific to every single cell. For creating tension maps, PSD was calculated for every pixel before fitting and extracting parameters.

### Measuring myomerger intensity and cluster detection

Freehand region of interest (ROI) drawn from myomerger-labeled cells at different timepoints was used to get mean intensity for surface myomerger estimation. For finding a region-wise correlation, the merging of live IRM and fixed fluorescence images of the same cell was necessary and performed by using the ImageJ (FIJI) template matching plugin. 24 × 24 pixels (1.72 × 1.72 µm^2^) FBR regions were chosen for measuring tension, and the same regions were then merged on the fluorescence sample to measure the mean intensity from a particular FBR. The probability density estimate of mean myomerger intensity per cell was plotted using the ksdensity function of MATLAB.

For detecting myomerger “clusters” from IF images, local thresholding was carried out first, followed by object detection using MATLAB. The mask of detected objects in multiple rectangular ROIs was used to identify pixels in the original image, and the mean intensity was noted as the mean intensity of the clusters of that region. The remaining pixels in the ROI were used to calculate the mean of the diffused background. ROIs were always chosen in regions inside the cell.

## Data Availability

The original contributions presented in the study are included in the article/supplementary material, further inquiries can be directed to the corresponding author. Further information and request for resources and reagents should be directed to and will be fulfilled by the lead contact, Bidisha Sinha (bidisha.sinha@iiserkol.ac.in).
