# Supplementary material for: Early tension regulation coupled to surface myomerger is necessary for the primary fusion of C2C12 myoblasts
Source: Front Physiol. 2022 Oct 14;13:976715. doi: 10.3389/fphys.2022.976715 (PMC7613732; doi:10.3389/fphys.2022.976715)
Supplement: Supplementary file 1 [file DataSheet1.PDF]

SUPPLEMENTARY MATERIAL

Early tension regulation coupled to surface myomerger is necessary for the primary fusion of C2C12 myoblasts

Madhura Chakraborty[1], Athul Sivan[1#], Arikta Biswas[1+], Bidisha Sinha[1]

[1]Department of Biological Sciences Indian Institute of Science Education and Research Kolkata, Mohanpur, Nadia – 741246, India
Email correspondence: bidisha.sinha@iiserkol.ac.in
#Present address: Institute of Cell Dynamics and Imaging, Von-Esmarch-Straße 56, 48149 Münster, Germany
+Present address: Mechanobiology Institute, National University of Singapore, 5A Engineering Drive, Singapore 117411

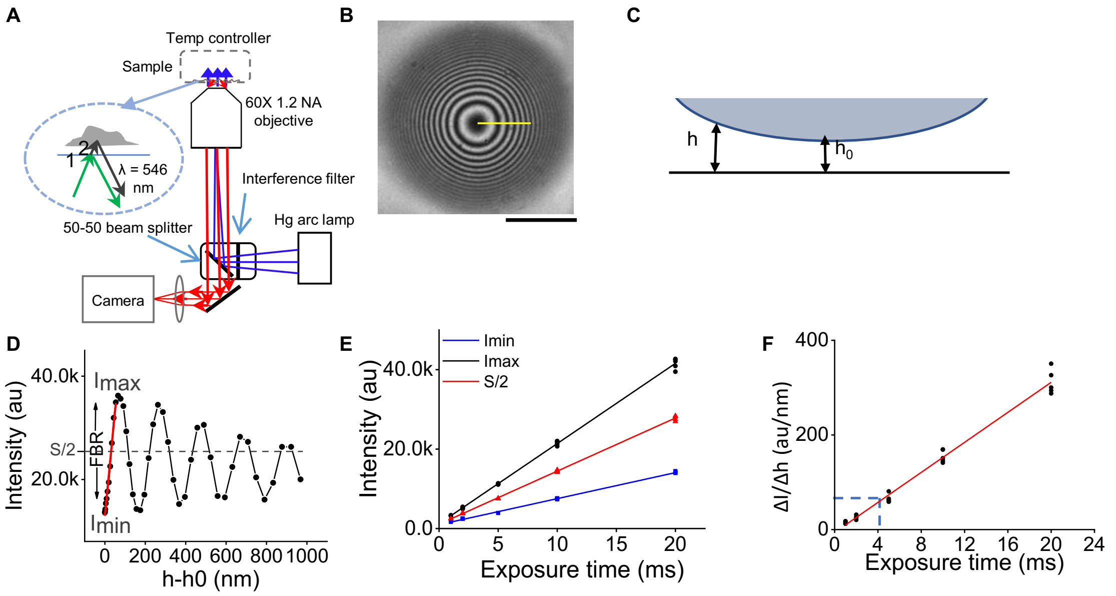

Figure S1: Schematic and calibration Interference Reflection Microscopy (IRM) techniques *(A) Schematic of IRM (B) IRM images of a 60 µm polystyrene bead attached on the glass surface showing interference pattern (C) Cross-sectional representation of bead attached on the glass surface displaying the profile of its distance from coverslip (height with h_0 being the reference and h- h_0 used as relative height. (D) Intensity vs relative height profile derived from the bead in (B) along the yellow line. Intensity minima (I_{min}), maxima (I_{max}) and S/2 (background intensity) were marked out. (E) Dependence of I_{min} , I_{max} and S/2 on exposure times (F) Line profile of slope (intensity to height conversion) vs exposure times. These calibrations are used to estimate the conversion to be used in images of cells. Cell's I_{max}, I_{min}, S/2 are compared against E and F to find the conversion factor to be used. Exposure time is altered to change the signal mimicking altered reflectivity* (Biswas, Alex and Sinha, 2017).

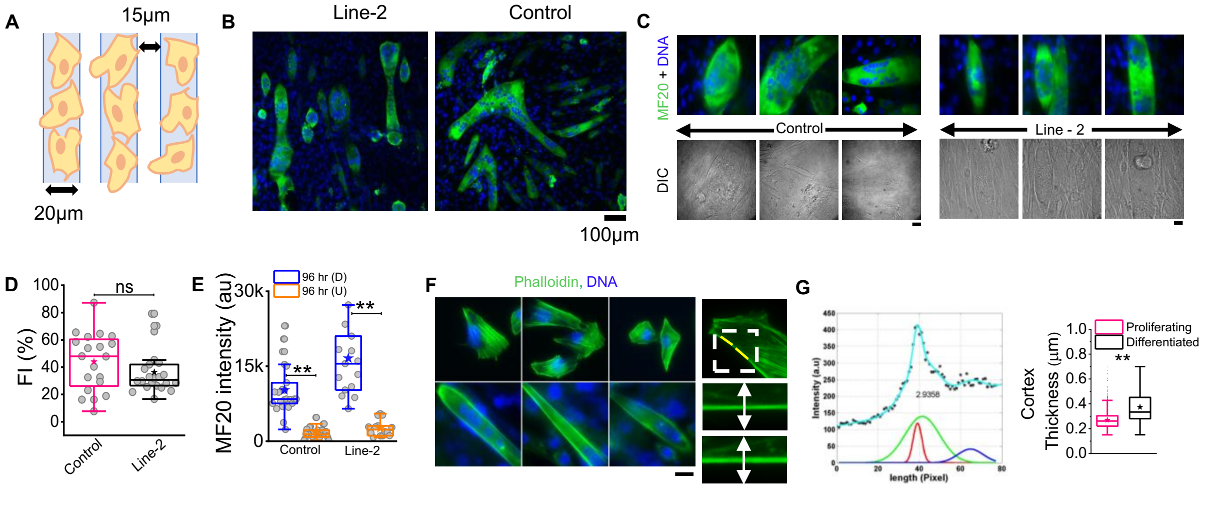

Figure S2: Fusion index and cortex thickness analysis *(A) Schematic of micropatterning on Line-2 micropatterns (B) MF20 labelling on samples differentiating on micropatterned coverslips (Line-2) and non-patterned coverslip (Control) (C) MF20 labelling of myotubes on 5th day of differentiation (96 hr D) and corresponding DIC images of the same (bottom panel) (D) Fusion index calculation; n = 40 independent frames used to measure 50 myotubes (E) Comparison of MF20 change in micropatterned and non-micropatterned substrates (F) Measurement of cortex thickness* (Kumar, Saha and Sinha, 2019) *involved confocal imaging of mononucleated cells (cells in GM) and myotubes labelled with Phalloidin to visualize F-actin. Typical region of interest (ROI) shown in yellow straightened line (80 x 77 pixels) and zoomed image of cortex straightened along such lines (G) a typical multi term gaussian fitting graph; 38 ROIs from 16 proliferating cells and 34 ROIs from 13 myotubes used to compare cortex thickness. Scale bar = 20 µm.*

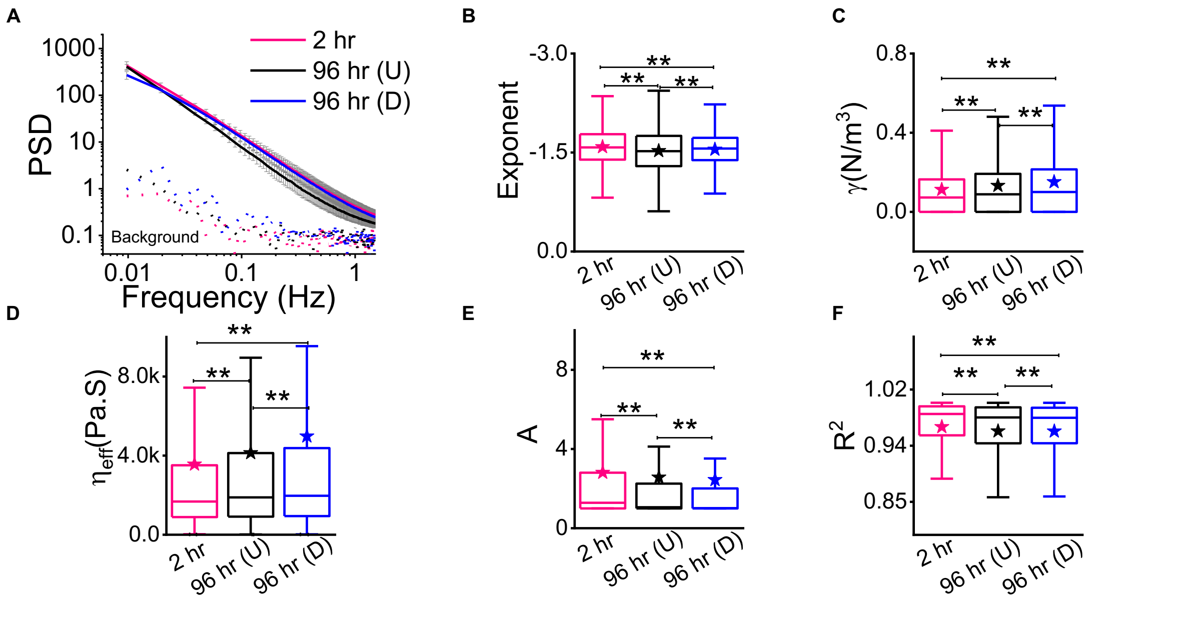

Figure S3: Power spectral density (PSD) and mechanical parameters extracted *(A) Comparison of PSDs from 2 hr, 96 hr (U) and 96 hr (D) cells (B) Exponent calculated from a linear fit to log (PSD) versus log(f)plot for frequencies ranging from 0.04 to 0.4 Hz. (C) Confinement (D) Effective cytoplasmic viscosity (E) Active temperature and (F) R^2 value from the fitted data. Cells and FBR numbers correspond to those in Figure 2. Mann-Whitney U statistical significance test is performed, ** denotes p value < 0.001.*

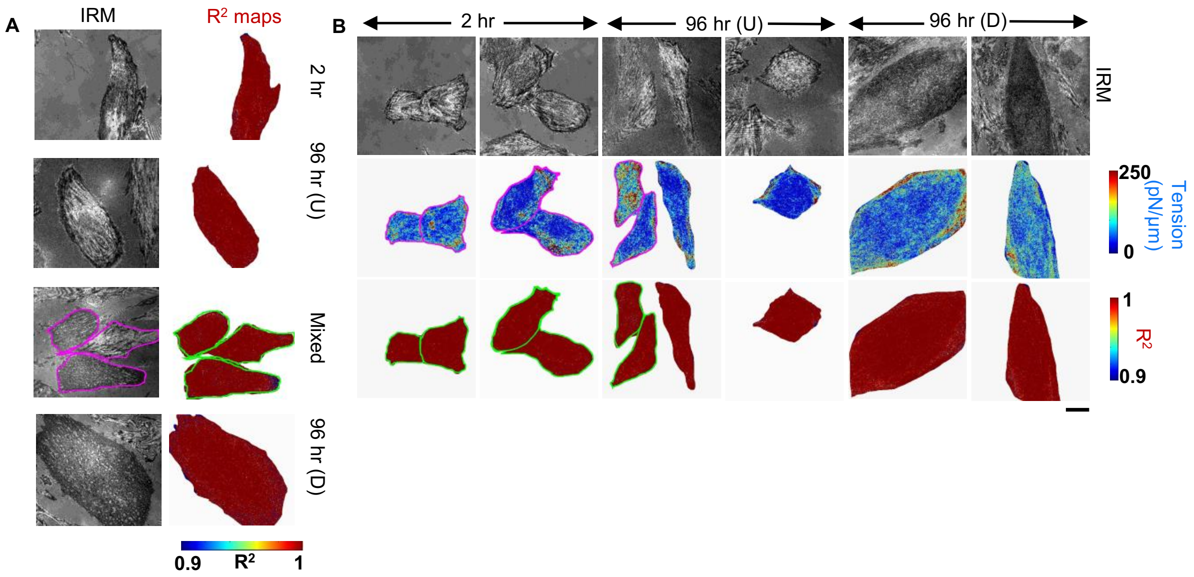

Figure S4: Tension and R^2 maps of three different population of cells *(A) Respective R^2 maps of tension mapped cells shown in Figure 3 (B) IRM images, and its corresponding tension and R^2 maps of 2 hr, 96 hr (U) and 96 hr (D) cells. Scale bar = 20 µm.*

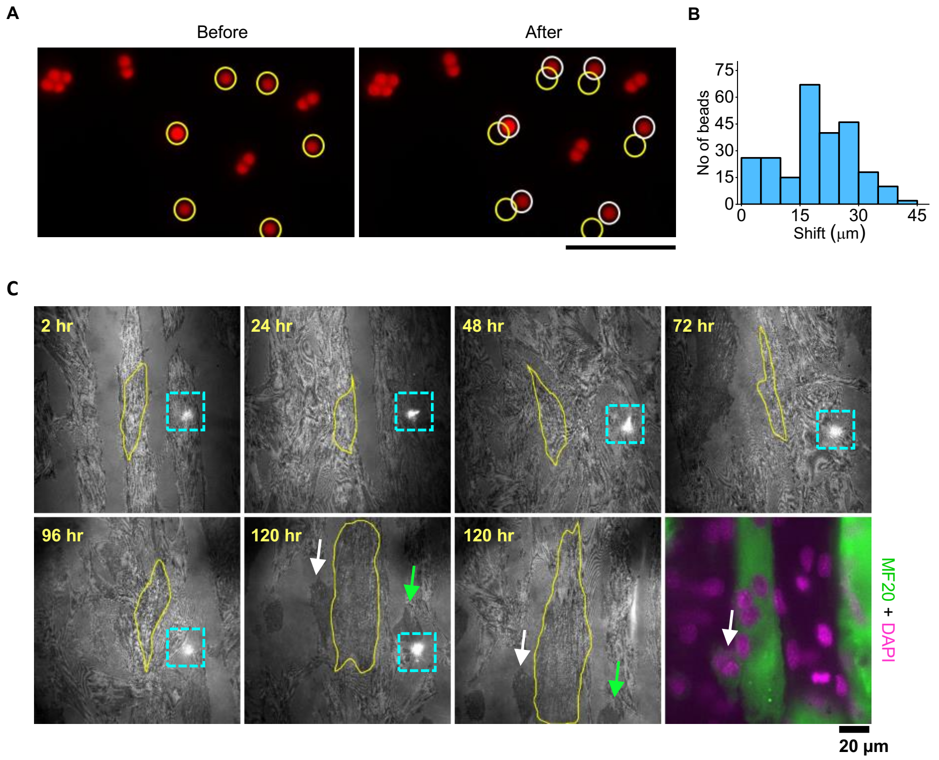

Figure S5: Tracking beads using transformation matrix *(A) Epifluorescence image of 2 µm beads from a particular image field with two different attempts together as before and after to assess displacement from centroids (B) Distribution of average shift measured for beads between consecutive attempts of any particular experiment; n = 4 independent experiments used to obtain 41 beads with 16 different attempts (C) Same cell tracking successfully using transformation matrix, cyan boxes in each day's image denoted the same artifacts to understand the same place, green arrows in last two frames showing upper and lower portion of same myotube which finally labelled by MF20. Scale Bar = 20 µm.*

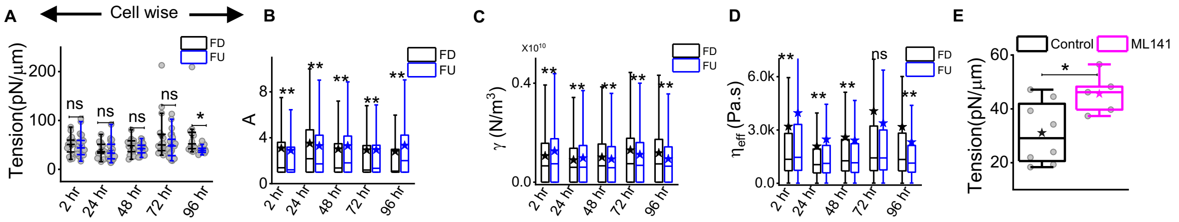

Figure S6: Cell-wise comparison and other mechanical parameters comparison from single-cell tracking *(A) Membrane tension comparison between FD and FU cells across time points (B) Active temperature comparison (C) Confinement comparison (D) Effective cytoplasmic viscosity comparison; Numbers of cells and FBRs are similar as in Figure 5. (E) Cell-wise tension comparison between control and ML141 treated C2c12 cells in growth media. Mann-Whitney U statistical significance test is performed, ** denotes p value < 0.001.*

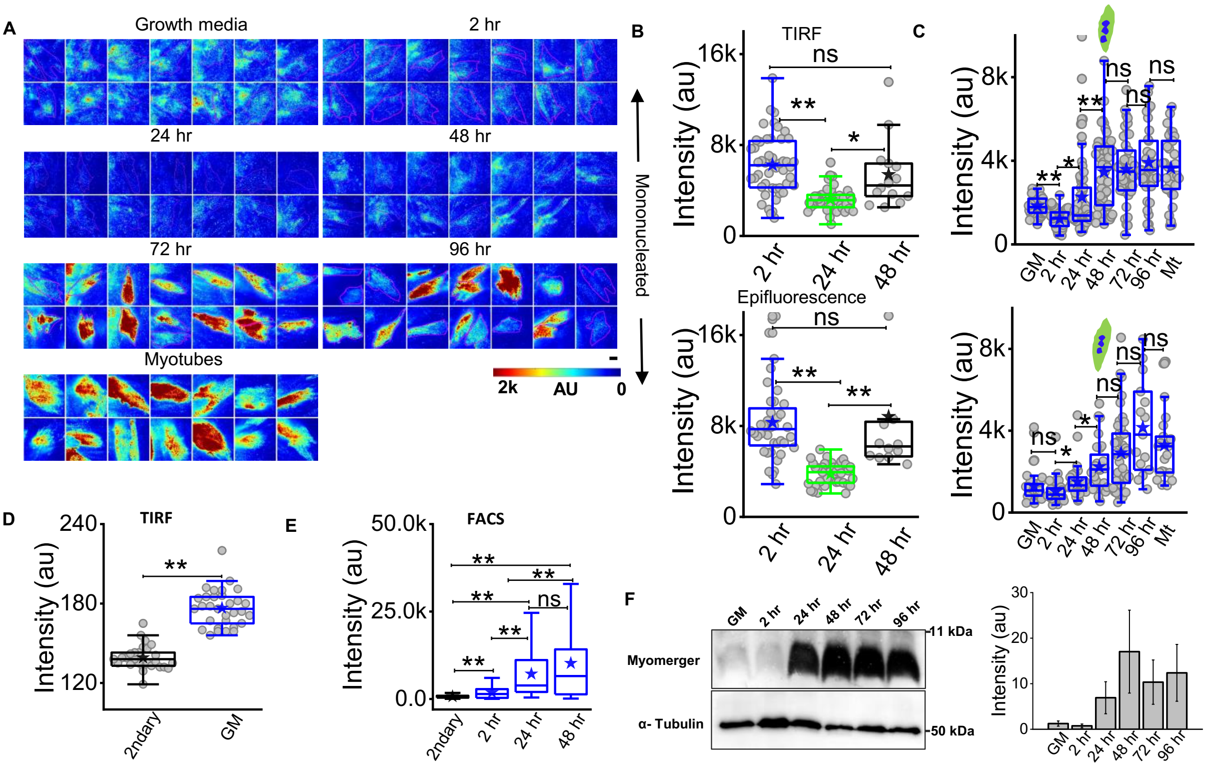

Figure S7: Heterogenous expression of myomerger at different time points *(A) Cell surface expression of myomerger shown as heatmaps from immunostaining data from a representative single-set (B) Comparison of myomerger intensity of a particular set measured using TIRF (top) and epifluorescence (bottom) displaying similar trends n = 1 try used to get 48 cells (2 hr), 43 cells (24 hr) and 13 cells (48 hr) (C) Surface myomerger intensity comparison of different time points for two sets which shown myotubes at 48th hr (D) Secondary signal comparison in TIRF (E) FACS data showing myomerger expression at different time points including secondary-only control (F) Western blot showing whole cell expression of myomerger at different time points and its intensity comparison. Cells from different time points were scraped in cold PBS and then centrifuged at 3500 rpm for 5 min, cell pellet were then lysed using lysis buffer (mixture of RIPA and protease inhibitor cocktail) for 45 min in ice cold condition. Lysed cells were sonicated for 5 seconds with 30 seconds interval for 6 cycles. The whole cell lysate (WCL) then centrifuge in 4° C at 3500 rpm for 10 min. The whole cell lysate was then collected as supernatant. The WCL was then used first for estimating the total protein concentration with Bradford assay. Same protein amounts were loaded in SDS PAGE well. NuPage loading dye along with β-marcaptoethanol was used as a loading buffer. Before loading, samples were incubated with loading buffer at 37° C for 30 min. 0.2 μm PVDF membrane was used for transferring of protein from gel*

*to membrane. After transfer membrane kept in blocking solution using 5% skimmed milk in TBST (Tris buffer saline with 0.1 % tween 20) for 3 hr. Primary antibody for myomerger (Anti-sheep ESGP antibody 1:500 dilution) and Alpha-Tubulin (Anti-rabbit Alpha tubulin 1:20000 dilution) were added after blocking and kept at 4°C shaker for overnight. Secondary antibody for respective proteins (Rabbit Anti-sheep IgG- AP and Goat Anti-Rabbit HRP) were then added after several washing steps with TBST buffer. Secondary treatment was done for 2 hr. Finally blots were visualized using chemidoc system. Scale bar = 10 µm, Mann-Whitney U statistical significance test is performed, ** denotes P value < 0.001, * denotes P value < 0.05 and ns denotes not significant.*

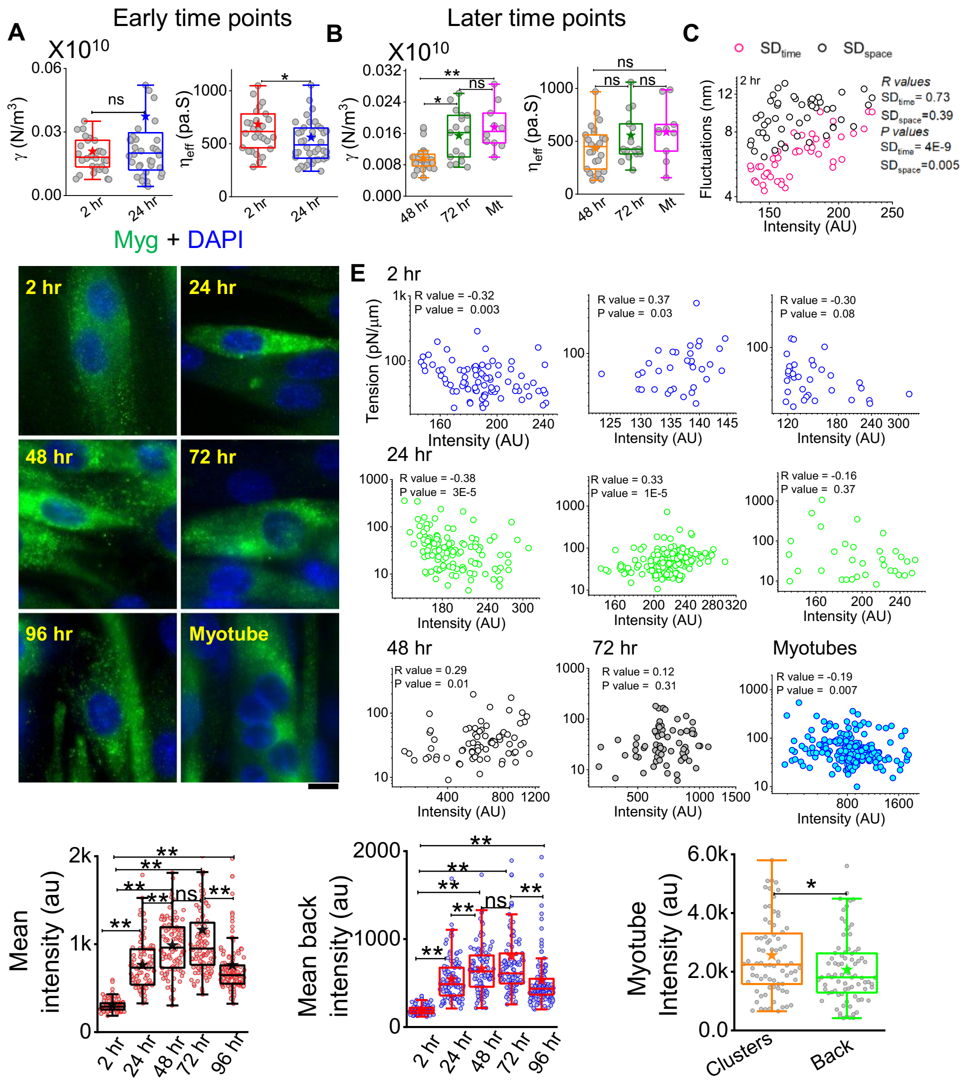

Figure S8: Other mechanical parameters, local correlation and myomerger cluster-to-background ratio. *(A, B) Confinement and effective viscosity - fitting parameters of fbr-wise fluctuations data used to derive tension in Figure 7. Mann-Whitney U statistical significance test is performed, ** denotes p value < 0.001, * denotes p value < 0.05 and ns denotes not significant.(C) Correlation of fluctuations with myomerger intensity at the same region – for typical regions of different samples. (D) Images of myomerger IF at different time points (E) Correlation of tension with intensity at other typical regions (F) left: Mean intensity of clusters detected in IF images of myomerger at different time points; centre: mean intensity of the diffused background around clusters; right: mean intensity of clusters and diffused background for myotubes. Satistics of data is same as for Figure 8G.*

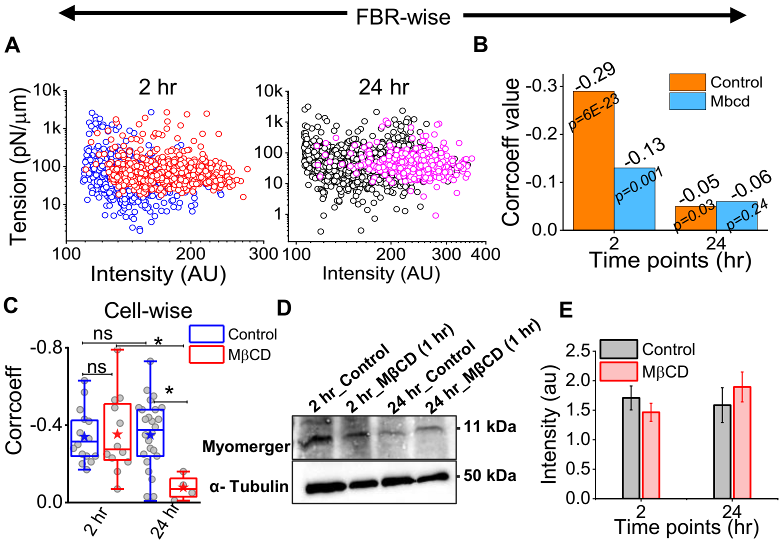

Figure S9: Effect of cholesterol depletion on correlation coefficient. *(A) Typical scatter plot for intensity and tension from FBRs for single cells at 2 hr (left) or 24 hr (right) in control (blue/black) or MβCD-treated condition (pink/red). Note that these are not same cells before and after cholesterol depletion (B) Correlation coefficients and p-values for scatter plots shown in (A). (C) Box-plot comparison of correlation coefficients of those scatters that show negative correlation (even if ns). The reduced coefficient is more prominent for 24 hr (D) Western blot showing the expression pattern of myomerger upon MβCD treatment (E) Intensity of myomerger from WB.*

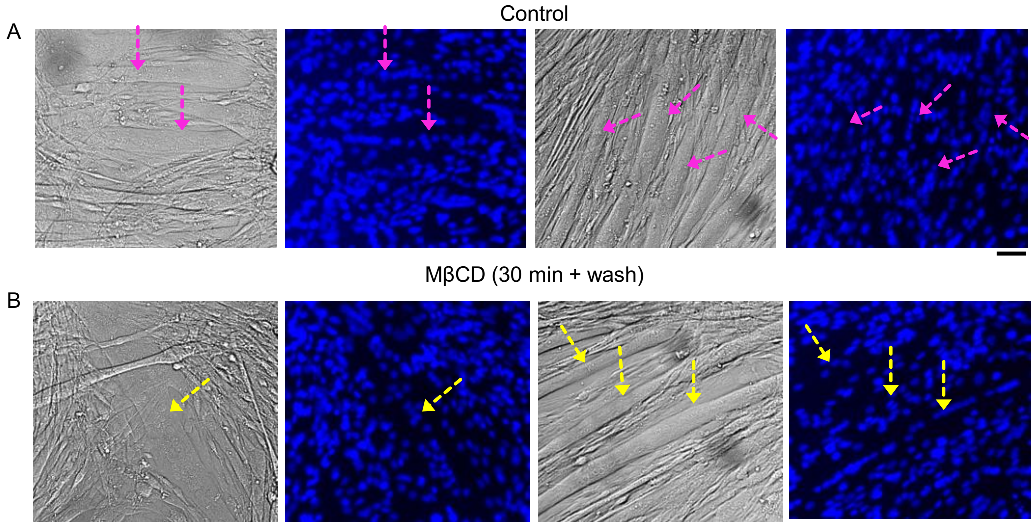

Figure S10: Effect of pulsed cholesterol depletion on fusion. *(A) DIC (grey and DAPI staining (blue) for control cells after 96 hr of DM treatment (B) DIC (grey and DAPI staining (blue) for cells treated with MβCD at 24 hr but only for 30 min after which media is replaced by DM again. Images are after 96 hr of DM treatment. Scale bar = 50 µm.*

Table S1: List of parameters measured and reported in plots in figures.

P values are calculated using Mann-Whitney unless mentioned tohave been also calculated using Linear Mixed-effect Model (LMM)

**Figure 1D (left)**								
**Parameters**	**Condition**	**N_{cells}**	**n_{rois}**	**Mean**	**SD**	**SEM**	**Median**	**P value**

	Myoblasts	32	1526	138	35	6.13	131	
MF20 intensity	96 hr (U)	25	626	1834	1406	281.26	1659	4.00E-10
	96 hr (D)	24	1647	10128	4831	1007.35	8545	1.00E-08
**Figure 2E (left)**								
**Parameters**	**Condition**	**N_{cell}**	**N_{FBR}**	**Mean**	**SD**	**SEM**	**Median**	**P value (wrt 2hr)**
	2hr	52	947	4.3	0.9	0.03	4.3	
SD time (nm)	96 hr (U)	40	712	3.7	0.7	0.03	3.7	0*
(12x12								
pixels)	96 hr (D)	32	948	3.4	0.9	0.03	3.4	0**
**Figure 2E (right)**								
**Parameters**	**Condition**	**N_{cell}**	**N_{FBR}**	**Mean**	**SD**	**SEM**	**Median**	**P value (wrt 2hr)**
SD space (nm)	2hr	52	947	7.8	2	0.07	7.6	
(12x12 pixels)	96 hr (U)	40	712	6.6	1.5	0.05	6.4	0
	96 hr (D)	32	948	5	1.3	0.04	5	0
**Figure 2G (left)**								
**Parameters**	**Condition**	**N_{cell}**	**N_{FBR}**	**Mean**	**SD**	**SEM**	**Median**	**P value (wrt 2hr) (LMM)**
Tension (pN/µm)	2hr	52	14269	259	746	6.3	61.9	
(4x4 pixels)	96 hr (U)	40	20090	327	834	5.9	82	0.6
	96 hr (D)	32	35041	398	1043	5.6	90.2	0.01
**Figure 2G (right)**								
**Parameters**	**Condition**	**N_{cell}**	**N_{FBR}**	**Mean**	**SD**	**SEM**	**Median**	**P value (wrt 2hr)**
Tension (pN/µm)	2hr	52	14269	259	746	6.3	61.9	
(4x4 pixels)	96 hr (U)	40	20090	327	834	5.9	82	<0.001
	96 hr (D)	32	35041	398	1043	5.6	90.2	<0.001
**Figure 3B**								
**Parameters**	2hr	52	52	1.2	0.16	0.024	1.19	
SD (SD_{time})	96 hr (U)	40	40	1	0.17	0.028	0.99	1.8E-5
(nm)	96 hr (D)	32	32	0.77	0.15	0.025	0.76	2.7E-13
	96 hr (D)	32	32	0.77	0.15	0.025	0.76	2.7E-13
**Figure 3C**								
**Parameters**	**Condition**	**N**	**n**	**Mean**	**SD**	**SEM**	**Median**	**P value (wrt 2hr)**
SD (SD_{space})	2hr	52	52	0.28	0.06	0.008	0.28	
(nm)	96 hr (U)	40	40	0.3	0.07	0.012	0.28	0.376
	96 hr (D)	32	32	0.24	0.05	0.008	0.26	0.007
SD (Tension)	**Condition**	**N**	**n**	**Mean**	**SD**	**SEM**	**Median**	**P value (wrt 2hr)**
(pN/µm)	2hr	52	52	681	414	59.81	605	
	96 hr (U)	40	40	787	248	40.75	740	0.005
	96 hr (D)	32	32	877	375	65.2	817	0.002
**Figure 4C**								
**Parameters**	**Condition**	**N_{cell}**	**n_{FBR}**	**Mean**	**SD**	**SEM**	**Median**	**P value (wrt 2hr)**
	**(Finally differentiated)**							
Tension (pN/µm)	2hr	1	2563	171	408	8.07	47.6	

	24hr	1	2560	157	491	9.72	36.23	0
	48hr	1	2037	197	419	9.28	50.18	0.183
	72hr	1	3053	338	684	12.39	74.35	1.00E-41
	96hr	1	2209	264	656	13.95	73.2	3.00E-31
SD $_{time}$ (nm)	2hr	1	3385	6.01	1.36	0.023	5.91	
	24hr	1	6239	7.07	2.03	0.025	6.89	2.00E-142
	48hr	1	6798	6.34	1.2	0.014	6.26	3.00E-32
	72hr	1	6711	4.78	0.71	0.008	4.78	0
		1	5089	4.44	0.71	0.009	4.38	0
**Figure 4D**								
**Parameters**	**Condition**	**N_{cell}**	**n_{FBR}**	**Mean**	**SD**	**SEM**	**Median**	**P value (wrt 2hr)**
	**(Finally undifferentiated)**							
Tension (pN/µm)	2hr	1	1342	193	453	12.38	46.69	
	24hr	1	560	314	774	32.74	74.29	6.00E-12
	48hr	1	2346	173	489	10.09	45.3	0.493
	72hr	1	2750	193	495	9.44	54.99	0.0012
	96hr	1	2997	152	446	8.15	43.13	0.009
**Parameters**	**Condition**	**N_{cell}**	**n_{FBR}**	**Mean**	**SD**	**SEM**	**Median**	**P value (wrt 2hr)**
	**(Finally undifferentiated)**							
SD $_{time}$ (nm)	2hr	1	3020	6.63	1.53	0.027	6.58	
	24hr	1	2638	5.25	1.4	0.028	5.08	0
	48hr	1	4615	5.34	1.47	0.021	5.11	0
	72hr	1	6172	5.52	1.43	0.018	5.53	0
	96hr	1	7118	6.36	1.71	0.02	6.17	0
**Figure 5A**								
**Parameters**	**Condition**	**N**	**n**	**Mean**	**SD**	**SEM**	**Median**	**P value (wrt 2hr)**
	**(FD+FU)**							
Tension	2hr	24	24	62.12	16.12	3.29	57.31	
(pN/µm)	24hr	24	24	49.32	14.22	2.9	48.8	0.006
	48hr	24	24	50.03	10.37	2.12	48.92	0.004
	72hr	24	24	68.31	39.64	8.09	53.75	ns
	96hr	13	13	67.38	45.36	12.58	49.86	ns
**Figure 5B**								
**Parameters**	**Condition**	**N**	**n**	**Mean**	**SD**	**SEM**	**Median**	**P value (wrt**
	**(Finally differentiated)**							
Relative	2hr	16	16	1.22	0.26	0.07	1.29	
SD $_{time}$ (nm)	24hr	16	16	1	0.18	0.04	1.05	0.001
	48hr	16	16	0.92	0.24	0.06	0.82	ns
	72hr	16	16	0.84	0.19	0.06	0.78	ns
	96hr	12	12	1.22	0.27	0.07	1.28	0.01
	**(Finally undifferentiated)**							
Relative	2hr	24	24	1.18	0.3	0.06	1.14	
SD $_{time}$ (nm)	24hr	24	24	1.02	0.2	0.04	0.97	ns
	48hr	24	24	0.99	0.22	0.04	0.98	ns
	72hr	24	24	1.02	0.22	0.05	0.98	ns
	96hr	17	17	1.18	0.3	0.06	1.14	ns
**Figure 5C**								
**Parameters**	**Condition**	**N**	**n**	**Mean**	**SD**	**SEM**	**Median**	**P value (wrt 2hr)**

	**(Finally differentiated)**							
Relative	2hr	12	12	1	0	0	1	
Tension (pN/μm)	24hr	12	12	0.8	0.24	0.07	0.75	0.003
	48hr	12	12	0.9	0.19	0.05	0.89	ns
	72hr	12	12	1.3	0.66	0.19	1.11	ns
	96hr	8	8	1.6	1.05	0.37	1.26	ns
	**(Finally undifferentiated)**							
Relative	2hr	12	12	1	0	0	1	
Tension (pN/μm)	24hr	12	12	0.9	0.33	0.09	0.96	ns
	48hr	12	12	0.8	0.19	0.06	0.76	ns
	72hr	12	12	0.9	0.38	0.11	0.95	ns
	96hr	5	5	0.7	0.24	0.11	0.68	0.02
**Figure 5G**								
**Parameters**	**Condition**	**N_{Cell}**	**n**	**Mean**	**SD**	**SEM**	**Median**	**P value (wrt control)**
Fusion Index (%)	Control	16	16	39.54	11.98	2.99	39.35	
	ML141	16	16	15.09	11.65	2.91	10.71	4.00E-05
	MβCD	16	16	0	0	0	0	3.00E-07
Tension	Control	12	9	34.27	15.74	5.25	28.78	
(pN/μm)	MβCD	12	15	53.99	12.58	3.25	55.88	0.007
**Figure 5H (left)**								
**Parameters**	**Condition**	**N_{Cell}**	**n_{FBR}**	**Mean**	**SD**	**SEM**	**Median**	**P value (wrt 2 hr)**
	**(Finally differentiated)**							
SD_{time}	2 hr	12	82893	5.57	1.81	0.00629	5.26	
(nm)	24 hr	12	57773	6.78	1.91	0.008	6.61	0
	48 hr	12	85871	5.38	1.42	0.005	5.24	0
	72 hr	12	66018	5.63	1.7	0.007	5.51	0
	96 hr	8	91448	5.07	1.42	0.005	4.93	0
	**(Finally undifferentiated)**							
SD_{time}	2 hr		87626	5.73	1.74	0.006	5.59	
(nm)	24 hr		71161	6.46	1.89	0.007	6.27	0
	48 hr		109153	6.36	1.72	0.005	6.21	0
	72 hr		91307	5.79	1.68	0.005	5.66	0
	96 hr		28950	6.35	1.71	0.01	6.21	0
**Figure 5H (right)**								
**Parameters**	**Condition**	**N_{Cell}**	**n**	**Mean**	**SD**	**SEM**	**Median**	**P value (wrt 2 hr)**
	**(Finally differentiated)**							
Tension	2 hr		33007	229.29	664.91	3.18	53.33	
(pN/μm)	24 hr		24452	168.19	463.52	2.96	41.4	0
	48 hr		22179	227.41	668.59	4.49	51.42	0.005
	72 hr		35653	321.78	951.79	5.04	65.32	4.00E-111
	96 hr		40465	279.45	754.11	3.75	64.06	5.00E-107
	**(Finally undifferentiated)**							

Tension	2 hr		34726	256.62	726.9	3.9	63.03	
(pN/µm)	24 hr		25030	208.86	568.45	3.593	49.33	0
	48 hr		39562	186.82	497.79	2.502	47.003	0
	72 hr		39910	231.81	696.72	3.487	55.39	0
	96 hr		11450	160.36	448.96	4.196	42.64	0

Figure 6C

Parameters	Condition	n	Mean	SD	SEM	Median	P value (wrt 2 hr)	
Myomerger	GM	52	546	173	24.05	553	1.00E-06	
TIRF	2 hr	50	400	94.15	13.31	395		
	24 hr	52	312	96.15	13.33	313	4.00E-05	
	48 hr	51	444	132	18.55	431	0.10969	
	72 hr	53	906	527	72.47	788	5.00E-09	
	96 hr	51	766	512	71.75	668	0.003	
	Myotubes	20	1149	607	135.94	1169	2.00E-06	

Figure 7B and 7C

Parameters	Condition	n	Mean	SD	SEM	Median	P value	
Myomerger Intensity (au)	2 hr	32	203	35.44	6.26	202		
	24 hr	31	375	183	33.01	357	5.00E-04	
Tension (pN/µm)	2 hr	32	44.19	24.26	4.29	38.89		
	24 hr	31	30.79	13.98	2.51	27.53	0.0025	
Myomerger intensity (au)	48 hr	38	497	241	39.24	443		
	72 hr	25	1076	947	189	755	6E-4 (wrt 48 hr)	
	Myotubes	10	818	859	271	504	0.15885 (wrt 48 hr)	
Tension (pN/µm)	48 hr	38	44.7	61.89	10.04	31.76		
	72 hr	25	67.86	63.12	12.62	49.09	0.0014 (wrt 48 hr)	
	Myotubes	10	45.27	14.44	4.57	44.67	0.038 (wrt 48 hr)	

Figure 8B (left)

Parameters	Condition	N_{cells}	Mean	SD	SEM	Median	P value	
							(wrt control)	
Intensity (au)	Control_2 hr	11	135	8.05	2.43	133.79		
	MβCD_2 hr	9	173	32.57	10.85	173.34	0.001	
	Control_24 hr	10	212	24.26	7.67	215.38		
	MβCD_24 hr	8	235	33.43	11.82	241.92	ns	

Figure 8B (right)

Parameters	Condition	N_{cells}	Mean	SD	SEM	Median	P value	
							(wrt control)	
Tension (pN/µm)	Control_2 hr	11	49.76	19.34	5.83	45.89		
	MβCD_2 hr	9	75.98	30.49	10.16	70.03	0.02	
	Control_24 hr	10	45.16	32.35	10.23	33.35		
	MβCD_24 hr	8	46.69	18.39	6.5	42.03	ns	

Figure 8G

Parameters	Condition	$N_{clusters}$	n_{rois}	Mean	SD	SEM	Median	P value
								(wrt 2 hr)
Cluster ratio	2 hr	90	10	1.56	0.17	0.02	1.53	
	24 hr	100	10	1.51	0.15	0.01	1.5	ns
	48 hr	100	10	1.54	0.19	0.02	1.51	ns
	72 hr	108	10	1.5	0.16	0.01	1.5	0.001
	96 hr	150	10	1.48	0.16	0.01	1.48	2.00E-16

Figure 8H

Parameters	Condition	$N_{clusters}$	n_{rois}	Mean	SD	SEM	Median	P value
								(wrt 2 hr)
Cluster numbers	2 hr	90	10	1.04	0.21	0.022	1.04	
	24 hr	100	10	0.99	0.32	0.031	1.02	ns
	48 hr	100	10	0.91	0.24	0.024	0.93	6.00E-05
	72 hr	110	10	0.72	0.26	0.025	0.76	0.001
	96 hr	150	10	0.73	0.24	0.02	0.75	
	Myotubes	80	10	0.33	0.29	0.032	0.27	2.00E-16

Figure 8J (right)

Parameters	Condition	$N_{clusters}$	n_{rois}	Mean	SD	SEM	Median	P value
								(wrt control)
Cluster numbers	Control_2 hr	30	10	0.43	0.15	0.03	0.43	
	MβCD_2 hr	30	10	0.34	0.22	0.04	0.31	ns
	Control_24 hr	39	10	1.31	0.07	0.01	1.29	
	MβCD_24 hr	49	10	1.24	0.08	0.01	1.23	5.00E-07

Figure 8J (left)

Parameters	Condition	$N_{clusters}$	n_{rois}	Mean	SD	SEM	Median	P value
								(wrt control)
Cluster ratio	Control_2 hr	29	10	1.36	0.09	0.01	1.35	
	MβCD_2 hr	28	10	1.24	0.07	0.01	1.23	2.00E-06
	Control_24 hr	39	10	1.31	0.07	0.01	1.29	
	MβCD_24 hr	49	10	1.24	0.08	0.01	1.23	1.00E-08

Figure S2B

Parameters	Condition	n	Mean	SD	SEM	Median	P value (wrt 2 hr)	
Fusion Index	Myotubes	50	30.75	13.27	1.88	28.23		

Figure S3

Parameters	Condition	n	Mean	SD	SEM	Median	P value (wrt 2 hr)	

Figure S3B

Exponent	2 hr	42815	-1.58	0.33	0.0016	-3.55		
	96 hr (U)	40148	-1.52	0.34	0.0017	-2.86	3.00E-162	
	96 hr (D)	85129	-1.55	0.25	0.0008	-2.86	2.00E-77	

Figure S3C

γ (N/m^3)	2 hr	14269	0.112	0.149	0.0012	0.073		
	96 hr (U)	20090	0.133	0.175	0.0012	0.089	6.00E-18	
	96 hr (D)	35041	0.151	0.199	0.001	0.1	5.00E-60	

Figure S3D

η_{eff} (Pa.s)	2 hr	14269	3560	7261	60.79	1676		
	96 hr (U)	20090	4138	8249	58.2	1886	7.00E-14	
	96 hr (D)	35041	4981	12998	69.44	1969	3.00E-40	
**Figure S3E**								
A	2 hr	14269	2.81	3.01	0.025	1.28		
	96 hr (U)	20090	2.57	2.94	0.02	1.06	0	
	96 hr (D)	35041	2.45	2.89	0.015	1.01	0	
**Figure S3F**								
R^2	2 hr	14269	0.96	0.046	4.00E-04	0.98		
	96 hr (U)	20090	0.96	0.05	4.00E-04	0.98	0	
	96 hr (D)	35041	0.96	0.05	3.00E-04	0.98	0	
**Figure S4B**								
**Parameters**	**Condition**	**n**	**Mean**	**SD**	**SEM**	**Median**	**P value (wrt 2 hr)**	
	**(Finally differentiated)**							
A	2 hr	33007	3.08	3.14	0.017	1.38		
	24 hr	24452	3.5	3.16	0.02	2.17	3.00E-99	
	48 hr	22179	3.01	3.08	0.021	1.36	0.07	
	72 hr	35653	2.93	3.07	0.016	1.19	9.00E-12	
	96 hr	40465	2.8	3.03	0.015	1.09	0	
	**(Finally undifferentiated)**							
A	2 hr	34726	2.88	3.02	0.016	1.2		
	24 hr	25030	3.29	3.16	0.02	1.72	3.00E-64	
	48 hr	39562	3.29	3.14	0.016	1.81	1.00E-95	
	72 hr	38873	2.95	3.03	0.015	1.34	2.00E-04	
	96 hr	11450	3.33	3.07	0.029	2.01	9.00E-75	
**Figure S4C**								
**Parameters**	**Condition**	**n**	**Mean**	**SD**	**SEM**	**Median**	**P value (wrt 2 hr)**	
	**(Finally differentiated)**							
γ (N/m^3)	2 hr	33007	3.08	3.14	0.017	1.38		
	24 hr	24452	3.5	3.16	0.02	2.17	3.00E-99	
	48 hr	22179	3.01	3.08	0.021	1.36	0.07	
	72 hr	35653	2.93	3.07	0.016	1.19	9.00E-12	
	96 hr	40465	2.8	3.03	0.015	1.09	0	
**Parameters**	**Condition**	**n**	**Mean**	**SD**	**SEM**	**Median**	**P value (wrt 2 hr)**	
	**(Finally undifferentiated)**							
γ (N/m^3)	2 hr	34726	2.88	3.02	0.016	1.2		
($X10^{10}$)	24 hr	25030	3.29	3.16	0.02	1.72	3.00E-64	
	48 hr	39562	3.29	3.14	0.016	1.81	1.00E-95	
	72 hr	38873	2.95	3.03	0.015	1.34	2.00E-04	
	96 hr	11450	3.33	3.07	0.029	2.01	9.00E-75	
**Figure S4D**								
**Parameters**	**Condition**	**n**	**Mean**	**SD**	**SEM**	**Median**	**P value (wrt 2 hr)**	
	**(Finally differentiated)**							
η_{eff} (Pa.s)	2 hr	33007	3194	8422	46.36053	1351.2		
	24 hr	24452	2081	5105	32.64666	1051.05	0	
	48 hr	22179	2587	5088	34.17101	1265.9	4.00E-13	

	72 hr	35653	4066	12227	64.75784	1427.9	9.00E-12	
	96 hr	40465	3177	7797	38.76384	1340.6	ns	
	**(Finally undifferentiated)**							
η_{eff} (Pa.s)	2 hr	34726	3958	10257	55.04	1461		
	24 hr	25030	2475	7053	44.58	1119	0	
	48 hr	39562	2420	5528	27.79	1156	0	
	72 hr	38873	3387	7821	39.67	1427	0.01	
	96 hr	11450	2318	4760	44.49	1137	0	

Supplementary References

Biswas, A., Alex, A. and Sinha, B. (2017) ‘Mapping Cell Membrane Fluctuations Reveals Their Active Regulation and Transient Heterogeneities’, *Biophysical Journal*. doi: 10.1016/j.bpj.2017.08.041.

Kumar, R., Saha, S. and Sinha, B. (2019) ‘Cell spread area and traction forces determine myosin-II-based cortex thickness regulation’, *Biochimica et Biophysica Acta - Molecular Cell Research*, 1866(12), p. 118516. doi: 10.1016/j.bbamcr.2019.07.011.
